# Theoretical Perspectives on the Minimal and Narrative Self in the Schizophrenia Spectrum: An Integrative Review

**DOI:** 10.1002/wcs.70023

**Published:** 2026-02-16

**Authors:** Florestan Delcourt, Henry R. Cowan, Jordan Sibéoni, Mélissa C. Allé, Andreas C. R. Rasmussen, Rosa Ritunnano, Anne Giersch, Fabian Lo Monte, Jérôme Englebert, Bernard Pachoud

**Affiliations:** ^1^ Centre de Recherches Psychanalyse, Médecine et Société (CRPMS), École Doctorale Recherches en Psychanalyse et Psychopathologie (ED450) Université Paris Cité Paris France; ^2^ Department of Psychology Michigan State University East Lansing Michigan USA; ^3^ Service Universitaire Intervention Précoce en Psychiatrie : Soins – Entourage – Adolescents (IP2SEA), Centre Hospitalier Argenteuil Argenteuil France; ^4^ Epidemiology and Clinical Statistics for Tumor, Respiratory, and Resuscitation (ECSTRRA) Team, Saint‐Louis Research Institute (Inserm UMR 1342) Université Paris Cité Paris France; ^5^ CNRS, UMR 9193, Sciences Cognitives et Sciences Affectives (SCALab) Université de Lille Lille France; ^6^ Mental Health Center Amager University of Copenhahen Copenhagen Denmark; ^7^ Department of Clinical Medicine University of Copenhahen Copenhagen Denmark; ^8^ Center for Youth Mental Health University of Melbourne Melbourne Australia; ^9^ Institute for Mental Health, School of Psychology University of Birmingham Birmingham UK; ^10^ INSERM U1329 Strasbourg Translational Neuroscience and Psychiatry (STEP) Team Psychiatry Université de Strasbourg Strasbroug France; ^11^ Pôle de Psychiatrie, Centre Hospitalier Régional Universitaire de Strasbourg Strasbourg France; ^12^ Université Libre de Bruxelles Bruxelles Belgium; ^13^ Université de Liège Liège Belgium; ^14^ Université Catholique de Louvain Ottignies‐Louvain‐la‐Neuve Belgium

**Keywords:** meaning‐making, narrativity, phenomenology, psychosis, systematic review

## Abstract

The self and its disorders in schizophrenia have been studied extensively over recent decades. Much of this literature is grounded in a bipartite understanding of the self, distinguishing the pre‐reflective, minimal self from the reflective, narrative self. However, few studies have systematically examined the links between disturbances at these two levels of self. This integrative review addresses this gap by analyzing both theoretical and empirical contributions. Three theoretical models are described. The Structural model posits that minimal self‐disorders hierarchically give rise to narrative self‐disturbances and the schizophrenia phenotype, with a primarily pathogenic focus. The Dialectical model emphasizes reciprocal interactions between minimal and narrative self‐disturbances, generating the schizophrenia phenotype with both pathogenic and salutogenic implications. The Contextual model highlights social, territorial, and biological dimensions of the self and its disorders in context. Empirical studies specifically addressing the mechanistic links between minimal and narrative self‐disturbances remain scarce and preliminary. Overall, the literature appears preliminary and occasionally speculative, yet it suggests several promising avenues for future research and clinically relevant applications.

This article is categorized under:

Philosophy > Consciousness
Psychology > Theory and Methods

## Graphical abstract

The links between the minimal self (our most basic sense of being) and the narrative self (our personal story and identity) in schizophrenia are still unclear. They may work in three ways: the minimal self affecting the narrative self, both influencing each other, or both shaped by the person's context.

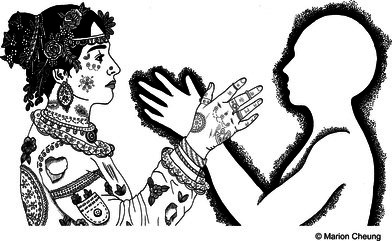

*I think that I think, and by thinking it I am*

*And I am because I think*

*And this sad knowledge confuses my science*

*Adds a night to my nights.*

*What am I without being and without a memory*

*Mixing tomorrow and yesterday*

*And unfolding within me this shifting moire*

*Dangerous as the sea.*

*Better for me is the sleep and its vague mixture#*

*Where I am burdened with nothing*

*And its dark theater whose play changes me*

*In a me no longer mine*.^1^

Jean Cocteau, *Je pense que je pense et d'y penser je suis* (in Clair‐Obscur).

[authors's translation]

## 1 Introduction

The self is a concept whose history is as extensive as its definitions are diverse. It appears across cultures from antiquity to post‐modernity and can be conceptualized through bodily experience, perception, thought, emotion, intention, identity, and morality, considered both individually and intersubjectively (Gallagher 2011; Kitcher 2021). Etymologically designating *what* enables the *I*, and referring to the processes that ground the constituent elements of *my* person (*Me*), the self has been examined throughout the history of psychiatry. Recent studies emphasize that it is neither monolithic nor static but instead exhibits a processual and patterned or architectonic organization (Basten and Touyz 2019; Daly et al. 2024; Fazakas et al. 2025; Gallagher 2011; Henriksen et al. 2021; Kircher and David 2003; Stanghellini et al. 2019; Thomsen, Cowan, and McAdams 2025). Over the last decades, this conception has supported a multilayered understanding of the self, composed of interacting levels and structures that appear disrupted in psychiatric disorders such as those within the schizophrenia spectrum. Historically and conceptually, schizophrenia has been described as a disorder profoundly affecting the self (Garrabé 1992; Jablensky 2010; Mishara et al. 2013), a view that also resonates with its Greek etymology (*σχίζω*, *skhízō* = to split; *φρήν*, *phrēn* = seat of emotions, mind, will). Thus, if schizophrenia involves a fundamental self‐disturbance (Sass and Parnas 2003), recovery‐oriented theorists have proposed that individuals may reappropriate aspects of the self and mitigate certain forms of self‐disturbance during recovery (Koenig 2016; Leamy et al. 2011; Murray and Menadue 2023; Thomsen, Cowan, and McAdams 2025; Thomsen et al. 2023).

The notion of the self in schizophrenia appears to involve two distinct levels: the pre‐reflective level (Sass and Parnas 2003), which ensures the tacit, a priori sense of the *I*, “of being a subject, a self‐coinciding center of action, thought, and experience” (Parnas et al. 2005), and the reflective, narrative level, which grounds the *Me* in an “internalized and evolving life story, integrating the reconstructed past and imagined future to provide life with some degree of unity and purpose” (McAdams and McLean 2013). The pre‐reflective level of consciousness—often described as a minimal, basic, or core self—refers to automatic and implicit processes, first‐personal manifestations of lived experience (such as embodiment, inner time‐consciousness, or minimal selfhood) that are understood as constitutive of more complex experiential levels involving focal objects of awareness such as perception, imagination, and memory (Gallagher and Zahavi 2008).

Conversely, the reflective, narrative level corresponds to a higher‐order mode of selfhood characterized by the capacity to interpret, articulate, and make meaning of experiences across time, through language and reflection. This mediated level of selfhood constructs identity through autobiographical narratives shaped by sociocultural context (McAdams and McLean 2013). These two levels of the self can also be traced in the work of various authors, including James (1890; I/Me), Sartre (1936; reflective/pre‐reflective consciousness), Damasio (1999; central‐/autobiographical‐self), as well as Gallagher (2000) and Zahavi (2010; minimal/narrative self). Researchers focusing on the pre‐reflective aspect of the self in schizophrenia and those examining its reflective aspect frequently refer to these levels as two poles of self‐experience.

Moreover, empirical studies in schizophrenia spectrum disorders have reported that pre‐reflective minimal self‐disturbances aggregate more strongly in these disorders than in other mental illnesses (Burgin et al. 2022; Fusar‐Poli et al. 2022; Henriksen et al. 2021; Nordgaard et al. 2021; Raballo et al. 2021). Likewise, several studies have identified disturbances at the narrative level of selfhood in schizophrenia (Berna et al. 2015; Conneely et al. 2020; Cowan et al. 2021; Fusar‐Poli et al. 2022; Harris et al. 2021; Lysaker, Kukla, et al. 2020; Lysaker, Minor, et al. 2020; Zhang et al. 2019).

Yet, while some authors have questioned, explored, and/or criticized the links between the minimal and narrative self (Belt 2019; Bortolan 2019; Gallagher 2000, 2013; Horváth 2024; Zahavi 2010, 2012), these connections remain unclear in research on the schizophrenia spectrum, despite the repeated use of this definition. Some studies (Ritunnano et al. 2022; Sabbah and Northoff 2024a; Svendsen et al. 2020) have suggested such links, but their precise relationships—understood broadly, for example, in theoretical or empirical terms—have not been systematically examined. This question is critical for deriving clinical insights from research on self‐experience, including elucidating the genesis of the disorder, understanding subjective knowledge of it, and identifying experiential barriers or resources that may support recovery. Given the lack of consensus regarding theoretical models or methodological approaches, we conducted an integrative review (Toronto and Remington 2020) to explore this issue in a systematic, exploratory, and descriptive manner: *what are the links between the minimal and narrative dimensions of the self in the schizophrenia spectrum?*

## 2 Method

An integrative review is a descriptive, *iterative*, systematic approach for exploring under‐researched, non‐standardized topics. It does not aim to measure effect sizes or assess efficacy, but rather to comprehensively *integrate* and synthesize diverse findings—theoretical or empirical—in order to generate an exploratory, holistic understanding of a given topic or phenomenon (Toronto and Remington 2020). Its purpose is to describe—rather than validate or assess performatively—the various trends in the literature related to a specific question. Accordingly, all types of peer‐reviewed papers are considered to capture the range of possible answers, directions of inquiry, and conceptual approaches to the research question.

We followed Toronto and Remington's *Step‐by‐Step Guide to Conducting an Integrative Review* (2020) to carry out the study. The research protocol was registered on PROSPERO (CRD42024483376), and a PRISMA flowchart was produced in accordance with the Preferred Reporting Items for Systematic Reviews and Meta‐Analyses (2020 version) guidelines.

A systematic literature search was conducted for published peer‐reviewed papers (excluding books and theses) in the PubMed, PsycINFO, PsycArticles, Web of Science, PBSC, and CINAHL databases, with no restrictions on publication date or paper type. Included languages were Danish, English, French, German, Italian, Norwegian, Portuguese, and Swedish; however, all articles were required to have an English‐language abstract for screening purposes. The initial search was conducted on October 29, 2023, and a supplemental search was performed to include papers published up to June 14, 2025. Bibliographic management was carried out using Zotero. The search terms were:

(Phenomenolog* OR Lived Experience* OR Anomalous Experience* OR Self Disturbance* OR Self Disorder* OR Basic Self OR Minimal Self OR Ipseity OR Life‐World) AND (Hermeneut* OR Identit* OR Autobiograph* OR Life Story OR Narrative Self OR Narrativ* OR Meaning* OR Interpretati*) AND (Schizophrenia Spectrum OR Schizo* OR Psychosi* OR Psychoti*)

Papers were included if they (1) addressed the minimal self; (2) AND the narrative self; (3) IN clinical disorders within the schizophrenia spectrum.

Papers were excluded if they (1) failed to distinguish between the minimal and narrative self; (2) AND/OR did not refer to the clinical schizophrenia spectrum population (i.e., only referring to nonclinical populations or other forms of mental disorder).

After extracting references from the databases, each title and abstract was independently screened by six reviewers (A.C.R.R., F.D., F.L.M., H.R.C., J.S., M.C.A.). This step involved dividing each study selection into three categories: *Excluded* (if the reviewer was certain of exclusion), *Included* (if the reviewer was certain of inclusion), and *In Doubt* (if the reviewer hesitated, even slightly, between exclusion and inclusion), based on the criteria outlined above. Screening decisions were determined by consensus among the research team. A second screening was then performed, in which all *Included* and *In Doubt* papers were re‐evaluated by all reviewers. Papers remaining in the *In Doubt* category were discussed by at least three reviewers to reach consensus on inclusion or exclusion. Subsequently, included papers were equally and individually assigned to reviewers (including BP, JE, and RR at this stage) for full‐text reading. At this stage, papers could still be excluded by consensus if they did not meet the inclusion criteria upon full‐text review.

Quality analysis of the papers was conducted by each reviewer using the Critical Appraisal Skills Programme (CASP) checklists during collective evaluation sessions. Theoretical studies were similarly assessed using the Quality Assessment Tool for Theory‐Based and Literature Review Studies (QATTL; Crawford et al. 2024), which evaluates both paper structure (e.g., abstract, introduction, framework) and supplementary elements (e.g., limitations, tables). Importantly, given the open‐ended and exploratory nature of an integrative review—aimed at examining non‐standardized or under‐researched literature or questions—the quality assessment of included papers did not influence their selection or inclusion. Rather, it served to descriptively highlight the characteristics of the entire corpus.

**FIGURE 1:**
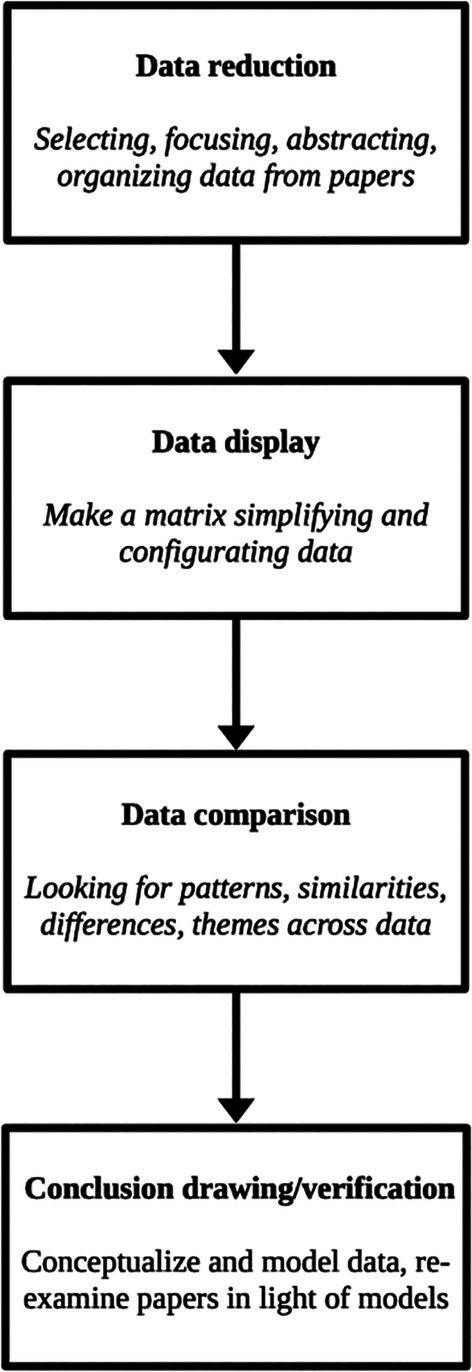
Constant Comparison Method flowchart.

Data were synthesized following Whittemore and Knafl's (2005, *in* Toronto and Remington 2020) four‐phase Constant Comparison Method (Figure 1). The first two phases were designed to select, simplify, and extract relevant data from the primary sources (data reduction) and to present them concisely, clearly, and graphically (data display). To this end, individual matrices were created, in which each reviewer, after reading each paper, recorded in each column: (1) the bibliographic reference, (2) the type of paper and/or methodology used, (3) its methodology, when applicable (i.e., sample, aim, and measures), (4) results and/or overall contributions and perspective, and (5) the reported links between the minimal and narrative dimensions of the self in the schizophrenia spectrum. All individual matrices were subsequently combined into a single final matrix, and collective sessions among reviewers were conducted to extract a descriptive model addressing the research question. The third phase (data comparison) involved identifying patterns, relationships, and themes that link the minimal and narrative dimensions of the self within the schizophrenia spectrum, with attention to plausibility, clustering, contrasts, common and unusual patterns, variabilities, potential contributing factors, and possible logical chains of evidence among the results. This modeling phase also included graphical representation of the findings. The final phase (conclusion drawing/verification) synthesized these links and confirmed their accuracy within the primary sources, highlighting theoretical and/or empirical conflicts and suggesting areas for further research.

## 3 Results

### 3.1 Description and Quality Analysis

**FIGURE 2:**
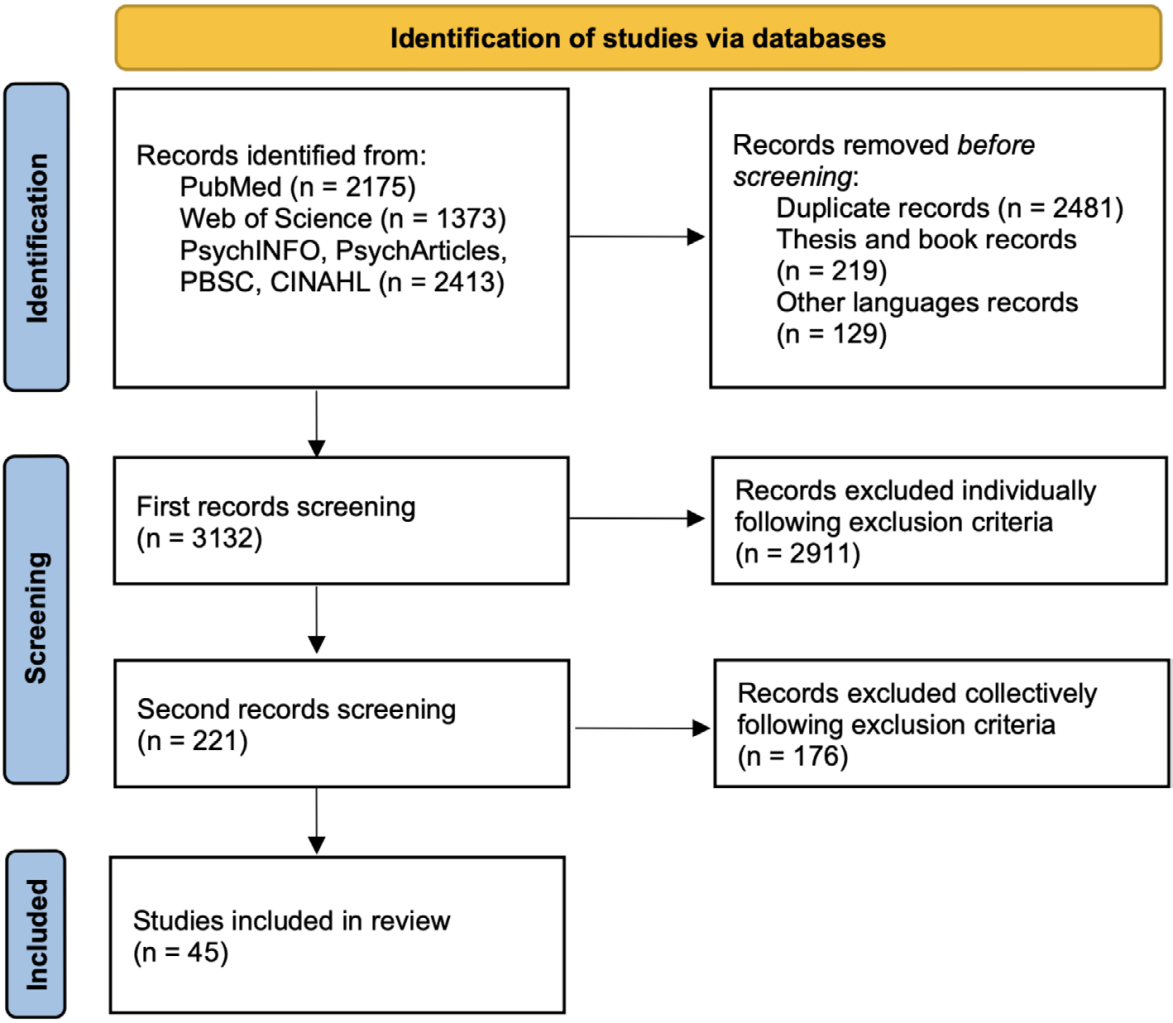
PRISMA flowchart.

After extracting 5961 references from the databases, we excluded 2481 duplicates, 129 references due to language, 219 references referring to books or theses, 2911 references following the first individual screening of titles and abstracts, and 176 references following the second collective screening of full texts (Figure 2). Of the 45 papers included (Table 1), the majority were theoretical or conceptual papers structured as narrative reviews (*n* = 28), with smaller proportions of case studies (*n* = 6), qualitative studies (*n* = 3), quantitative cross‐sectional studies (*n* = 4), quantitative case–control studies (*n* = 2), and systematic reviews (*n* = 2).

**TABLE 1:** Data matrix summarizing the included papers.

Author(s), date, *Title*	Type of paper	Methodology of the paper	Main perspective of the paper	Results as reported in the models
Chamond (1999): *The illegitimacy of time in schizophrenia: A phenomenological approach*	Narrative review and case study (Article)	**Sample:** Males with schizophrenia (*n* = 2)	A disorder in the synchronization of external and internal, lived time seems to generate phenomenological, identity, symbolic‐meaning making and existential disturbances in schizophrenia	**Sp:** Disturbance of the time constitutive of the continuity of experience can disturb lived time and of narrative, identity, meaning functions. It can generate delusion and an existential illegitimacy, an aprioric uninhabitability of self and world **Dp:** Disturbances of narrativity and meaning‐making can retroactively reinforce the aprioric sense of existential illegitimacy, of basic uninhabitability of self and world
Naudin et al. (2000): *Defining the acoustico‐verbal hallucination as a self‐awareness disoder*	Narrative review (Article)	N/A	Acoustico‐verbal hallucination is presented as an alteration of the *mineness* of experience, desynchronizing, following Damasio's model, the proto‐self from the central‐self and the autobiographical‐self	**Sp:** Basic disturbances of the self can affect higher levels of the self (e.g., narrative) via a disturbance of the *mineness* of experience and its temporal integration, culminating in schizophrenia symptoms (i.e., auditory hallucinations) **Dp:** Minimal disorders should always be understood through their interaction with the narrative‐reflective levels of the self. This can be seen as a lack of harmonization, possibly temporal harmonization, between these two levels of the self, which generate their own disorders in an isolated and mutually interactive way
Gallagher (2003): *Self‐narrative in schizophrenia*	Narrative Review (Chapter)	N/A	Self‐narration in schizophrenia is said to be impaired following a disorder of temporal integration of information, minimal self‐reference, encoding and retrieving of autobiographical memories and metacognitive abilities	**Sp:** The narrative self would be based on the following processes, where the minimal self is located, which schizophrenia would disturb, and its symptoms would tend to be generated by the disturbance of one or more of these processes: 1° temporal integration of information, 2° minimal self‐reference, 3° encoding and retrieving autobiographical memories, 4° reflective metacognition
Ballerini (2004): *Schizophrenia, autism, idionomia, dis‐sociality*	Narrative review (Article)	N/A	Autism is presented as the specific global core trait of schizophrenia, on the semeiological, psychopathological and phenomenological levels. This tends to make schizophrenia a disorder of the subject's social dimension (i.e., dis‐sociality, idionomy, ontological insecurity, antagonomy), which he strives to compensate for	**Sp:** Disorders of identity, both social and personal, and of meaning‐making result from autistic vulnerability and its related phenomenological disorders in schizophrenia **Cso:** The subject's socio‐cultural and institutional context may play a role in the genesis of this process
Bilheran et al. (2009): *“Hyperdating” and its defensive function in psychosis*	Narrative review and case study (Article)	**Sample:** female with schizophrenia (*n* = 1), female with bipolar disorder (*n* = 1)	Hyperdating (i.e., excessively dating life events) is said to be a mechanism that attempts, unsuccessfully, to compensate for the phenomenological and autobiographical disorders in psychosis	**Sp:** A disturbance of lived time specific to psychosis seems to induce a disturbance of narrativity, which would generate delusional symptoms as a defense and compensatory mechanism **Dp:** Hyperdating would make the disturbance of lived time livable, as a phenomenological defense mechanism retroacting on this minimal disturbance, but providing support for the delusional symptomatology
Lysaker and Lysaker (2010): *Schizophrenia and alterations in self‐experience: a comparison of 6 perspectives*	Narrative review (Article)	N/A	The first‐person perspective in schizophrenia is explored through six theoretical orientations (i.e., early psychiatry, existential psychiatry, psychoanalysis, phenomenology, psychosocial rehabilitation, and dialogical psychology), to extract affinities and differences	**Sp:** An initial minimal disturbance of the self seems to induce disorders of narrativity and metacognition, as well as of the sense of self and events. This can tendentially generate the schizophrenia phenotype
Heinz et al. (2012): *Construction and interpretation of self‐related function and dysfunction in Intercultural Psychiatry*	Narrative review (Article)	N/A	Self‐disorders are placed in socio‐cultural perspective (i.e., from Western concepts to Caribbean, African, South‐East‐Asian societies), and while the pre‐reflective aspect seems universal, the reflective aspect seems more circumstantial	**Sp:** Disorders of the minimal self would disturb the narrative functions of the self and thus generate the schizophrenia phenotype **Cso:** Disturbances of the minimal self are always situated on the socio‐cultural ground and the usual ways of expressing one's experiences. This implies the need to contextualize these disturbances in the subject's shared and common sense
De Vries et al. (2013): *Self‐Disturbance in Schizophrenia: A Phenomenological Approach to Better Understand Our Patients*	Narrative review and case study (Article)	**Sample:** two male and two female with schizophrenia (*n* = 4)	The minimal self‐disorders in schizophrenia seem to be associated with disorders of perceptual experience of the world and narrative identity	**Sp:** Disturbances of the minimal self can induce a disturbance of identity, and these two disturbances of the self can induce the schizophrenia phenotype, in particular delusions, specific to each person and their way of living‐with these experiential disturbances
Mishara and Fusar‐Poli (2013): *The phenomenology and neurobiology of delusion formation during psychosis onset: Jaspers, Truman symptoms, and aberrant salience*	Narrative review (Article)	N/A	Following the work of Karl Jaspers, the formation of delusion arises not only from neurological conditions yet to be determined, but also from phenomenological disorders experienced by persons and their socio‐cultural and personal ways of understanding these experiences	**Sp:** A minimal self‐disturbance may disturb the development of identity and meaning‐making, and the interaction between these two disturbances, possibly via the processes of temporal continuity of experience and identity, would generate the delusion in schizophrenia **Dp:** Disturbances of the narrative‐reflective self can retroact on the minimal self through a loss of natural evidence, a feeling of perplexity in relation to self and world. This would generate symptoms and psychosocial disturbances **Cte:** Disturbances of the minimal self, notably through their supposed neurobiological origins and subsequent narrative‐reflective disturbances, would generate aberrant saliencies in the subject's environment **Cbi:** Some neurobiological factors have been and are still being considered as the cause of these disturbances of the self, especially minimal, and in connection with the narrative self (i.e., the dopaminergic pathway, the abnormal striatal dopamine firing in early psychosis)
Stanghellini et al. (2013): *Person‐centered psychopathology of schizophrenia: building on Karl Jaspers' understanding of patient's attitude toward his illness*	Narrative review (Article)	N/A	Following the work of Karl Jaspers, the person‐centered approach to caring for persons with schizophrenia is theorized as reopening the active, interpersonal possibility of meaning‐making about oneself and one's lived experiences	**Sp:** The schizophrenia phenotype is the result of the subject's efforts to understand and be agentic in coping with the disturbances of the minimal self, which are primarily given to the subject and can disturb the narrative‐reflective self‐functions **Dp/Ds:** The understanding modalities of the subject living pre‐reflective anomalous experiences can modulate it positively, through the active and meaningful process of personal recovery, or negatively, resulting in the schizophrenia phenotype defined as a passive and depersonalized position in relation to disturbances in the subject's experience, which can increase them **Cso/Cte:** Personal recovery, as a way of regaining an active and retroactive position on the phenomenological experience of the disorder, is a process involving a renewed connection with the social and everyday life of each subject
Nelson et al. (2013): *Is basic self‐disturbance in ultra‐high risk for psychosis (‘prodromal’) patients associated with borderline personality pathology?*	Quantitative study (Article)	**Sample:** persons with UHR of psychosis (*n* = 42; 21 males; mean age = 19.22 years old) **Aim:** to investigate the association between basic self‐disturbance and borderline personality pathology with UHR patients **Measures:** EASE, SCID‐II PQ‐BPD	UHR persons have disorders of minimal self‐experience (EASE) uncorrelated with narrative self‐disorders found in borderline personality (SCID‐II PQ‐BPD)	**Sp:** The lack of correlation, in a population of subjects at high risk of psychosis, between EASE and SCID‐II PQ‐BPD scores would indicate a structural, and possibly generative, anteriority of minimal self‐disorders over narrative self‐disorders
Mishara et al. (2013): *Self‐disturbances in schizophrenia: history, phenomenology, and relevant findings from research on metacognition*	Narrative review (Article)	N/A	The relevance and aspect of the concept of self‐disorders (*Ichstörungen*) is detailed historically and at present, in the context of the study and care of persons with schizophrenia, and related to metacognitive abilities and clinical practice	**Sp:** The idea that disturbances of the minimal self are prior to and generative of disturbances of the narrative‐reflective self and the schizophrenia phenotype seems to benefit from historical and current anchorage and support, particularly with regard to meta‐cognitive processes **Dp/Ds:** The way these disturbances are expressed and understood tends to modulate them. Indeed, it is through narrative‐reflective, and therefore meta‐cognitive, processes that these disorders are experienced and accessed, both socially and individually **Cbi:** If the connections between the minimal self, its disorders and neurological circuits are not yet clear, those relating to the neurocognitive bases linked to metacognition in the modulation of these disorders are clearer, despite their inherent heterogeneity
Moe and Docherty (2014): *Schizophrenia and the sense of self*	Quantitative study (Article)	**Sample:** Persons with schizophrenia (*n* = 50; 25 males; mean age = 39.09 years old), bipolar disorder (*n* = 17; 10 males; mean age = 37.24 years old) and group control (*n* = 14; 13 males; mean age 38.22 years old) **Aim:** to assess how *Sense of Self* is affected by schizophrenia compared to other psychotic disorders **Measures:** ASD, GAF, PANSS	Some aspects of the sense of self, from the pre‐reflective to the reflective level, seem particularly and specifically disturbed in schizophrenia, namely those linked to agentivity and the relationship with other persons	**Sp:** Schizophrenia symptomatology results from disorders of the minimal self and their repercussions on narrative‐reflective, sense‐making functions of the self, and above all through a disturbed agentivity
Chamond (2017): *Explaining, understanding and interpreting human experience: From hermeneutics to phenomenological anthropology*	Narrative review (Article)	N/A	Hermeneutics is presented through various mental operations and through the foundation of a liveable world, always already pre‐understood for the subject, and schizophrenia is presented as the disorder disturbing this ante‐predictive foundation	**Dp:** Psychosis, schizophrenia in particular, would disturb the aprioric interaction between pre‐reflective experience and the modalities of understanding. Hence, this disturbed interaction would generate an uninhabitability of self and world
Koenig (2017): *Recovery in schizophrenia and sense of self*	Narrative review (Article)	N/A	The relevance and aspects of the concept of personal recovery are presented in themselves, in relation to schizophrenia, and compared to other theoretical orientations (i.e., psychoanalysis, phenomenology, neurocognition) in their person‐centered clinical possibilities	**Dp/Ds:** Disturbances of the self, minimal or not, are imbricated with the narrative, identitary and existential processes of the self. Thus, personal recovery could be an active way of retroacting on these disturbances to the self by reshaping existential, narrative, identity framework **Cso/Cte:** Personal recovery, schizophrenia and associated self‐disorders must always be situated socio‐cultural context and daily life of the subjects
Cermolacce et al. (2018): *Multiple Realities and Hybrid Objects: A Creative Approach of Schizophrenic Delusion*	Narrative review and case study (Article)	**Sample:** male with paranoid schizophrenia (*n* = 1), male with schizo‐affective psychosis (*n* = 1)	The relevance and aspects of the Multiple Reality Theory model are linked to schizophrenia delusion, which is seen as another lived reality alongside the socially shared reality, which can have Hybrid Objects, crossing both realities, and thus offering material for clinical observation and action	**Sp:** Each subject always experiences and shapes multiple lived realities (e.g., of delusion, work, family). This is phenomenologically based on a lived experience, and reflectively on a way of understanding, acting, being oneself in each reality. These processes are disturbed in this disorder, and these tend to generate and sustain the schizophrenia phenotype **Dp/Ds:** Narrative processes encompassing disturbed pre‐reflective experience tend to produce solipsistic meaning‐making. However, the care, taking its anchors in the subject's world‐view and its immediate environmental anchors, could retroact on this experience, giving rise to new dialogical and practical possibilities of understanding, socialized and open to the subject's existential freedom **Cso/Cte:** The way in which pre‐reflective disturbances are understood, and made to be understood, can modulate their donation to the subject and the position‐taking. Some objects in the environment can crystallize experiences and understandings, and act as clinical tools that socially mediate these, due to their socially shared aspect
Parnas and Henriksen (2019): *Selfhood and its disorders*	Narrative review (Chapter)	N/A	The self is presented structurally, from the minimal to the narrative level, with the former preconditioning the latter, and through the differential specificities that its disorders can have in some psychopathologies.	**Sp:** Compared with melancholia and personality disorders, schizophrenia is characterized by the prominence of disorders of the minimal self through the structure of ipseity, which can affect the narrative self
Charbonneau (2019): *Ipseity and Self‐Phenomenological and clinical approaches*	Narrative review (Article)	N/A	The concept of ipseity, extended from the pre‐reflective dimension (*ipse*) to the reflective dimension (*idem*) of human identity, is presented and highlighted for its specificity and connections in the understanding of psychotic disorders and other psychopathologies	**Sp:** The disturbance of the time constitutive of experience and identity tends to generate disturbances in identity‐idem (narrative self) through a disturbance in identity‐ipse (minimal self) specific to schizophrenia
Naudin et al. (2019): *Self and non‐self as a phenomenological issue for psychiatric experience*	Narrative review (Article)	N/A	The concept of self is placed in relation to that of alterity and world, of non‐self, both phenomenologically and narratively, and all these aspects are themselves linked to the understanding and clinical care and recovery of persons with schizophrenia	**Sp:** The narrative processes of the self‐emerge from its minimal processes, and schizophrenia disrupts identity via various disorders of this basic stratum **Ds:** The narrative processes of the self‐frame the minimal processes, socially and individually. Clinically and through personal recovery, this tends to give psychotherapy a retroactive effect on disturbed pre‐reflective experience, reopening the subject to a new existential space of freedom and autonomy through dialog and the re‐making of meaning **Cso/Cte:** Personal recovery always relies on the subject's social and verbal dimension (i.e., groups of belonging, shared narratives and words, concrete daily spaces) in its existential‐narrative overcoming of the disturbed pre‐reflective experiences
Zandersen and Parnas (2019): *Identity Disturbance, Feelings of Emptiness, and the Boundaries of the Schizophrenia Spectrum*	Narrative review and case study (Article)	**Sample:** female with borderline and schizotypal personality disorder (*n* = 1)	Despite some diagnostic affinities, disorders of the narrative self seem to be more closely related to borderline personality disorder, whereas disorders of the minimal self seem to be more closely related to schizophrenia, even though they can also, but only secondarily, disturb the narrative self	**Sp:** Disturbances of the minimal self impact the narrative identity dimension of the self, and thus generate structurally and differentially (i.e., compared to borderline disorder) the schizophrenia phenotype and experiences
Berkhout et al. (2019): *Identity, Subjectivity, and Disorders of Self in Psychosis*.	Qualitative study (Article)	**Sample:** service users with a FEP (*n* = 9; 4 males; mean age = 27.5 years old), family members of service users (*n* = 3) and carers of the clinic (*n* = 5) **Aim:** to explore the meanings, integration/rejection, experience of psychosis and antipsychotic medication from the perspectives of psychiatry service users in a FEP clinic, their physicians, case managers, other health professionals, and family members Measures: a critical qualitative ethnographic study of recorded in‐depth, longitudinal, open‐ended interviews and informal interviews	The socio‐cultural, embodied and enacted aspects of the self tend to be critical of the idealistic and disembodied perspectives of the self found in the literature on self‐disorders, following their importance in the formal genesis of these experiential disturbances. Indeed, they seem to remain linked to the circumstances of hospitalization, to the subject's narrative identity, with its associated emotions and meaning‐making, and to frictions with caregivers and healthcare system	**Dp:** Minimal self‐disorders are always linked to narrative identity, meaning‐making and emotions, and this non‐linear constant interaction generates psychotic lived experiences **Cso/Cte:** The minimal and reflective lived experience in psychosis is always linked to the relationship between the subject and its environment, both physical and socio‐institutional. However, these different aspects, and the very idea of the minimal‐narrative self, are culturally and socio‐politically circumstantiated, and this leads to the need to socialize self care and theory, and its phenotypic relations
Parnas and Zandersen (2020): *Phenomenology of a disordered self in schizophrenia: Example of an integrative level for psychiatric research*	Narrative review and case study (Chapter)	**Sample:** female with schizotypal and borderline personality disorder (*n* = 1) and with schizophrenia (*n* = 1)	Self‐disorders, traced historically and at present along minimal and narrative perspectives, seem to be specific to schizophrenia and to follow a process of integration, of anteriority of the minimal self over the narrative self, in the disturbances specific to this disorder that distinguishes it from other disorders, such as borderline personality disorder	**Sp:** Disturbances of the minimal self impact the identity and narrative dimension of the self, and thus structurally generate the schizophrenia phenotype and lived experience (e.g., the radical difference experience of *Anderssein* would be phenomenologically grounded and narratively resonant, but not grounded in the narrative self‐processes)
Svendsen et al. (2020): *Basic self‐disturbances are associated with Sense of Coherence in patients with psychotic disorders*	Quantitative study (Article)	**Sample:** 56 persons (28 males; mean age = 32.2 years old) with schizophrenia (*n* = 35), bipolar disorder (*n* = 13) and other psychoses (*n* = 8) **Aim:** to investigate the relationship between basic‐self‐disorders and sense of coherence in patients with psychotic disorders **Measures:** SOC‐13, EASE, SCI‐PANSS, GAF‐S, GAF‐F, SFS	The phenomenological disorders of the self (EASE) are negatively correlated with the narrative process of the sense of coherence (SOC‐13), and this, independently of the diagnosis, symptomatology or level of functioning of the persons	**Sp:** Disturbances of the minimal self are correlated with those of the sense of coherence, which refers to the narrative‐existential processes of the self, in particular through the sense of presence. This process may be prior to, but not unrelated to, the severity and the typicity of the schizophrenia phenotype
Pienkos (2020): *Schizophrenia in the World: Arguments for a Contextual Phenomenology of Psychopathology*	Narrative review (Article)	N/A	The contextual dimension, relating to interactions with the world, is highlighted and described in the phenomenological perspective, centered on the person and personal understanding capacities in the study and the care of persons with schizophrenia	**Sp:** Disturbances of the minimal self would have an impact on the meaning‐making process, which underpins the narrative and identity functions of the self. This could be done through hyper‐reflexivity, as a disorder of the integration of events experienced by the subject into a coherent narrative framework. This process tends to generate the schizophrenia phenotype **Dp/Ds:** Disturbances of the minimal self are always associated with concrete events and the way they are understood. This understanding tends to modulate the subject's experience and offers a more active role in recovery **Cso/Cte:** The social, daily, historic‐political context tends to modulate the subject's ability to understand the pre‐reflective dimension of lived events. This involves taking into account the subject's modalities of immersion, of navigation in the world in terms of norms and spaces
Rosen et al. (2021): *The Sensory and Perceptual Scaffolding of Absorption, Inner Speech, and Self in Psychosis*	Quantitative study (Article)	**Sample:** 117 persons (Mean age = 44.22 years old) with schizophrenia (*n* = 54; 29 males), bipolar disorder (*n* = 27; 13 males) and control group (*n* = 36; 19 males) **Aim:** to assess the phenomenology of sensory and perceptual experiences within the framework of absorption and inner speech with persons with psychosis compared to a non‐clinical control group **Measures:** PANSS, TAS, VISQ	The processes related to imaginary absorption and the emotional‐motivational‐dialogical tendency of subjects' inner speech are correlated with the positive symptomatology of psychotic disorders, in line with the idea of a basic disorder of consciousness	**Sp:** Disturbed sensory‐perceptual integration of the environment is associated with disorders relating to the minimal self (e.g., solipsistic/intense inner speech, absorption in imagination). This tends to have repercussions on the subject's meaning‐making and memory abilities, linked to the narrative self, and to positive symptomatology **Cbi:** The *Core Network Node* could be related to this minimal state of imaginative detachment of the subject from his environment, which may tend to influence his meaning‐making modalities and the symptomatology
Troubé (2021): *Exploration of Everydayness in Schizophrenia: A Phenomenological Approach*	Narrative review (Article)	N/A	The concept of *everydayness* is presented in its phenomenological aspect, as a pre‐reflective framework of experience, following the work of Bruce Bégout, and as a dimension particularly impaired in schizophrenia, which could be cared through narrative psychotherapy	**Ds:** Narrative psychotherapy could enable us to regain an existential‐reflective ascendancy modulating *everydayness* disorders (i.e., structure constituted by and constituting experience in a given meaning that is neutral, adjustable, repetitive, shared) **Cte:** The minimal and narrative‐reflective self are imbricated in the process of *everydaying* and the structure of *everydayness*, constituted by‐constituting natural attitude/familiar places that schizophrenia would disturb
Englebert (2021): *The “Territorial self”: Theoretical proposals from a phenomenological understanding of schizophrenia*	Narrative review (Article)	N/A	The concept of the territorial self, acting immediately in the environment always given to the subject, is discussed, related to the minimal and narrative aspects of the self, and put into perspective, in its nosographic specificity, in the understanding and care of persons with schizophrenia	**Sp:** Compared with other psychotic disorders, schizophrenia is characterized by a disturbance of the minimal and territorial self (i.e., which interacts with the environment and other persons, and manifests our mundane way of being) **Ss:** Disturbances of the minimal and territorial self require pre‐reflective clinical mediations (e.g., dance, theater, art therapy) **Cte:** Some aspects of the minimal and narrative self are always linked to the territorial self. Moreover, schizophrenia would be distinguished from other psychoses by its disturbance of the territorial self, leading to the ethical, behavioral, socio‐relational disengagement of the subject
Ritunnano et al. (2022): *Subjective experience and meaning of delusions in psychosis: a systematic review and qualitative evidence synthesis*	Meta‐synthesis (Article)	**Sample:** 24 qualitative studies associated with 373 persons with psychosis (*n* = 373; 210 males; 271 with schizophrenia, 41 with bipolar disorder or psychotic depression, 4 with unspecified psychosis, 2 with unspecified personality disorder, 1 obsessive‐compulsive disorder and previously paranoid schizophrenia) **Aim:** to explore how do persons with delusions, in the context of psychosis, experience and interpret changes in their sense of self, world and meaning **Measures:** Meta‐synthetic analysis of the qualitative studies (with ENTREQ guidelines, a critical realist philosophical stance, the qualitative evidence synthesis based on Cochrane guidance and the RETREAT criteria) extracted with a systematic review protocol (Quality assessment via CASP and NICE checklists)	The delusional symptomatology, in pathogenesis or recovery, seems related to affective and linguistic dynamic processes between disorders of lived experience and the meaning‐making, from the subpersonal to the sociocultural level, involving: a rearrangement of the lived world merged with intense emotions; a doubting, losing, and finding oneself again process within delusional realities; a searching for meaning, belonging, and coherence beyond dysfunction	**Sp:** Various disturbances in pre‐reflective experiences result in disturbances in identity, meaning‐making and social relations. These disturbances are the basis for the emergence of delusions in psychotic disorders, particularly schizophrenia **Dp/Ds:** Basic disorders of experience always interact with the subject's life story, identity, social life and modality of understanding. This would induce a retroactivity of the narrative processes of the self on the pre‐reflective experience, and possibly thus generating these basic disorders, the associated symptomatology, and also the process of personal recovery **Cso/Cte:** Disturbances related to the minimal and narrative self, and their interactions, are always already situated on an environmental, political and social context. It is thus necessary to integrate these dimensions into the care
Lobaccaro (2022): *Default Mode Network, Schizophrenia, and Narrativity. Comments on Psychopathology of Language*	Narrative review (Article)	N/A	The DMN, implicated in the pathogenesis of schizophrenia, relates not only to linguistic processes, but to the phenomenological aspect of the self in this disorder, and to the intertwining of narrative and imaginative processes toward an imaginary desituation paralleling socially shared life, which tends to disrupt basic and meaningful interactions between the subject, other subjects and the world	**Dp:** Schizophrenia would be linked to the DMN, and not only via linguistics, but via narrative imagination (i.e., the meaning‐making process representing and shaping experiences), which is altered and altering the meaningful aprioric relation to self and world **Cso/Cte/Cbi:** The neural networks linked to the DMN would generate an intertwined disturbance of the minimal and narrative self, disturbing the aprioric meaningful presence to the socio‐environmental framework, sustaining a disengagement from shared concrete world
Ritunnano (2022): *Overcoming Hermeneutical Injustice in Mental Health: A Role for Critical Phenomenology*	Narrative review (Article)	N/A	The concept of hermeneutic injustice (i.e., intrinsic and extrinsic disturbances to the person's ability to understand and make sense of one's lived experience) is presented, and its relevance is highlighted in the person‐centered understanding and care of the persons with schizophrenia, their lived experience and their own active capacities for understanding	**Sp:** Factors intrinsic to schizophrenia (e.g., cognitive disorders) can make the subject's lived experience unspeakable and incomprehensible. This could affect its evolution and sense of identity legitimacy **Ds:** Clinical relationships, through their dialogic, meaning‐making and narrative openness, should enable us to re‐open lived experience to understanding, and to modulate it existentially in order to live it better. To this end, we need to stop systemic hermeneutic violence through radical empathy, social recognition of lived experiences **Cso:** The understanding modalities of the lived experience are based on the subject's social group, as these extrinsic factors modulate the subject's ability to understand, express and legitimize the lived experience, giving rise to an illegitimacy of the psychotic experience, and certain social factors impact this process (e.g., epistemic asymmetry, hermeneutic marginalization). This, in turn, can reinforce disorders in the subject's pre‐reflective experiences
Mitchell and Meehan (2022): *How art‐as‐therapy supports participants with a diagnosis of schizophrenia: A phenomenological lifeworld investigation*	Qualitative study (Article)	**Sample:** persons with schizophrenia (*n* = 15; 8 males; between 28 and 73 years old) **Aim:** to explore how the lived experience of art‐making interacts with characteristic lived experiences associated with schizophrenia **Measures:** Phenomenological lifeworld analysis combined with coding elements from open‐ended interviews	The practice of art therapy, here mediated by painting and drawing, as an *act* in itself seems to have clinical implications with persons suffering from schizophrenia, in the bodily, temporal, spatial, social and selfhood dimensions, by reconsolidating the minimal sense of self and of a home‐like environment (cf. studio‐based art groups), as well as meaning‐making capacities	**Ss:** The act of art‐therapy could have an impact on the minimal and narrative disorders of the self, by restoring the subject's lived time and inhabitable, expressible memories, a more spontaneous corporeity, a *home‐like*, meaningful and pre‐reflectively inhabitable spatiality. These various phenomenological improvements also seem to induce an improvement of a narrative‐reflective nature
Green and García‐Mieres 2022: *Construing journeys to recovery from psychosis: A qualitative analysis of first‐person accounts*	Qualitative study (Article)	**Sample:** 156 first‐person accounts about the period after first treatment for a diagnosed psychotic disorder or psychotic symptom **Aim:** to explore whether persons who had their first‐person account published in the academic journal Schizophrenia Bulletin construed medication and mental health treatment as salient and the salience of CHIME processes and other factors related to recovery **Measures:** Computerized and manual coding textual analysis	The first‐person accounts of persons with psychotic disorders published in *Schizophrenia Bulletin* illustrate aspects of the persons' care journey, their pre‐reflective experiences of self and the CHIME model of personal recovery, and highlight the social, meaning and identity centered dimension of subjective wellness in these disorders	**Sp:** Disturbances of the minimal self and the phenomenological experiences of the world can generate disturbances of the narrative self, identity and the existential orientation of the subjects who provide accounts of their experience **Ds:** Events experienced on a non‐pre‐ reflective level could orient the subject toward the process of personal recovery. This, in turn, tends to attenuate pre‐reflective disorders by making them more expressible, livable, manageable, meaningful, identifiable with oneself, one's identity, one's existence **Cso/Cte:** The identity and meaning‐making processes of personal recovery, making the relationship with oneself and world more inhabitable, are to be found in the subject's concrete daily and social life, the way one manages one's life and one's disorder's signs
Simões 2023: *Pathological self‐consciousness and human identity*	Narrative review (Chapter)	N/A	The self‐disorders, from their pre‐reflective to their reflective dimension, are presented in the study of psychotic disorders and altered states of consciousness, and this presentation tends to show the multiple, relevant and socio‐cultural aspect of the self	**Sp:** Disturbances of the minimal self are prior, more present than those of an identity, linguistic, narrative and reflective nature, and especially through self agentivity and activity **Cso:** As opposed to altered states of consciousness, disorders of the minimal self in schizophrenia is not integrated into the socio‐cultural framework, and those of the narrative self are not altered as in schizophrenia disorder
Benítez‐Burraco et al. (2023): *An evolutionary account of impairment of self in cognitive disorders*	Narrative review (Article)	N/A	Some neurological, cognitive and language aspects are evolutionarily related to the minimal and reflective‐narrative dimensions of the self, and presented/compared in diametrically opposed ways between schizophrenia‐ and autism‐ spectrum disorders	**Cbi:** Neural, cognitive and bio‐genetic circuits and evolutionary leads tend to separate the autism spectrum from the schizophrenia spectrum. There would be distinct phenomenological and narrative disorders of the self (i.e., related to super cross‐modality, attributed to the abnormally enhanced, disinhibited connectivity in the cortico‐subcortical brain networks in schizophrenia), and this may tend to generate the symptoms of the disorder and vivid imaginations
Stanghellini et al. (2023): *The person's position‐taking in the shaping of schizophrenic phenomena*	Narrative review (Article)	N/A	The symptomatology and phenomenology of schizophrenia are presented as the dialectical result of the subject's position‐taking (*Stellungnahme*) in relation to one's pre‐reflective and existential experiences, of one's efforts to understand and live‐with one's phenomenological disorders, and this framework therefore proposes to restore an active, rather than passive, position to the person in relation to the manifestations of one's disorder, for both clinical and ethical purposes	**Dp/Ds:** A constant dynamic interaction between the phenomenological and hermeneutic processes of the self seems to be constitutive of lived, and therefore psychotic, experience. The way in which we take a hermeneutic position toward the lived experience can shape its form. By giving the person an active position in relation to these disturbances, which can modulate one's experience and evolution, this presents a clinical and an identity‐related interest **Cso/Cte:** In pathogenesis, one may take a negative existential‐identitary position, similar to social detachment and/or from their own body
Pienkos (2024): *How Narrative Counts in Phenomenological Models of Schizophrenia*	Narrative review (Commentary)	N/A	The notion of the minimal self is discussed, and relativized in the light of the significance of the subject's narrative and social dimension in the genesis of the disorder and its recovery	**Ds:** Personal recovery nuances the hierarchical structural reading of the minimal self by emphasizing the importance of the narrative self, meaning‐making and existential orientation. These processes are said to be constitutive of lived experience, and therefore of the clinical factors we need to work with **Cso/Cte:** It is necessary to include the socio‐daily dimension of one's life, related to the narrative self, in the person‐centered care
Sabbah and Northoff (2024a): *Global neural self‐disturbance in schizophrenia: A systematic fMRI review*	Systematic review (Article)	**Sample:** 23 fMRI studies associated with persons with schizophrenia (*n* = 471; 308 males; mean age = 35.63 years old) **Aim:** to make a map of the neuroimaging data onto the three‐layer topography model of the self (interoceptive, exteroceptive, mental) alongside hyperactivation and hypoactivation in schizophrenia, with respect to healthy controls **Measures:** Systematic review of fMRI studies on PubMed following a PRISMA chart and analyzed with an MNI—TAL translator software	The self‐disorders, from the pre‐reflective to the reflective dimension and defined by its capacities for self‐reflection, self‐referentiality, and self‐agency, seem to be generated, in schizophrenia, by abnormal and global cerebral activities in different regions, both high and low level, and particularly concerning the Mental Self and the Exteroceptive Self	**Sp:** The disturbance of neurological circuits can disturb the minimal self. This can have an effect on the higher dimensions of the self (i.e., reflective and narrative), generating the disorder phenotype and disturbing the relationship between self and the immediate environment **Cte/Cbi:** Disturbances of the minimal and narrative self are said to have a neurological basis (i.e., abnormal activity related to the self can be observed in a variety of different regions ranging from higher‐order transmodal to lower‐order unimodal regions), which may have consequences for our relation to the environment. This may imply the need to think about them in terms of their relation to spatial interaction modes (i.e., disturbance of self/non‐self differentiation; disturbance of limbic and subcortical networks)
Jimenez and Green (2024): *Disturbance at the self‐other boundary in schizophrenia: Linking phenomenology to clinical neuroscience*	Narrative review (Article)	N/A	The pre‐reflective/structural and reflective/experiential aspects of the self are described in the neuroscientific literature on disorders of self‐other boundary in schizophrenia, and different mechanisms affecting brain and cognition seem to impact these	**Ds:** The various neurological and cognitive processes and regions highlighted could be used to develop therapies designed to restore the *sense of self*, which could remedy these various self‐disorders **Cbi:** The structural/pre‐reflective and experiential/reflective‐narrative aspects of the self‐other demarcation are separately disturbed, following specific mechanisms, but also collectively, through various disturbances at the temporoparietal junction. These disturbances of the self could generate and help us to understand the schneiderian first rank symptoms and the phenotype of schizophrenia
Poupart and Laigle (2024): *Is delusion a narrative? Psychosis and narrativity*	Narrative review (Article)	N/A	The delusion is presented in a narrative approach as a problematic attempt (given its incorrigible, inconsequential and paradoxical aspects), and following the work of Paul Ricoeur, to set in plot, to verbalize and to make meaning the phenomenological experiences associated with psychotic disorders	**Sp:** Disturbances in the pre‐reflective experience of self and world are originally given and have an impact on the reflective‐identitary functions, which attempt to make sense of this experience through delusional narrative **Dp/Ds:** Delusion can retroact on pre‐reflective experience of self and world, attempting to make them habitable, meaningful, less intense. Thus delusion can be seen as an attempt to make meaning of phenomenological experience through socially shared words and ideas that can be grasped in clinical interactions
Yeh et al. (2024): *Impact of minimal self disorders on naturalistic episodic memory in first‐episode psychosis and parallels in healthy individuals with schizotypal traits*	Quantitative study (Article)	**Sample:** Person with a FEP (*n* = 10; 4 males; mean age = 19.8) and group control (*n* = 35; 14 males; mean age = 22.1) **Aim:** to investigate the modulation of the episodic memory of naturalistic events by the minimal self in FEP by using an immersive virtual reality paradigm and procedure **Measures:** WAIS‐IV, TMT B‐A, MAIA‐2, TSCS‐2, BPRS‐E, PANSS, NSSE, SOFAS, SPQ‐BR, Medication (Chlorpromazine equivalent dose)	Following a virtual reality immersion procedure manipulating the visuo‐motor synchronicity of a virtual avatar in events presented within a simulated everyday environment, and the assessment of minimal and narrative levels of the self in persons with FEP, it seems that embodiment is related to episodic memory, possibly through the level of schizotypy	**Sp:** Schizotypy is correlated with minimal and narrative self‐disorders, and spatial representation of the body is correlated with meta‐memory and the source of monitoring. This may show that minimal self‐disorder could underlie the meta‐cognition and insight disorders, which are related to narrative self **Cte:** At the basis of the disturbance of the link between the minimal and narrative self would lie a disturbed lived delimitation between self and world, with the pre‐reflective environment
Cowan (2024): *Aligning phenomenology and neuroscience of the basic and narrative self in schizophrenia*	Narrative review (Commentary)	N/A	Some neurological and cognitive processes, in particular those linked to the DMN, could be specifically involved in disorders of the phenomenological and narrative‐reflective levels of the self in schizophrenia, on each of these levels in a localized way or as a whole in a global way	**Sp:** A DMN disturbance could underlie a minimal self‐disturbance, and the latter, impacting this network, could generate narrative self‐disturbances **Cbi:** The narrative and phenomenological dimensions of the self seem to be aligned, both structurally and pathogenically, and in particular through disorders of a broader set of DMN, for the narrative self, and of a specific set of this network, for the minimal self
Sabbah and Northoff (2024b): *Basic self‐disturbance in schizophrenia: From neuronal to mental topographic dedifferentiation*	Narrative review (Commentary)	N/A	Some neurological and cognitive disorders seem to induce a global disturbance of the self in schizophrenia, minimal and/or narrative, and of the demarcation between the self and the non‐self, its environment, and these disturbances seems to be similar to a neuronal‐mental topographic dedifferentiation	**Sp:** The disturbance of some neurological circuits can disturb the minimal self. This can have an effect on the higher processes of the self (i.e., reflective‐narrative), generating the phenotype of the disorder and disturbing the relationship between self and environment **Cte/Cbi:** Disturbances of the minimal and narrative self may have a neural basis (i.e., abnormal activity related to the self can be observed in a variety of different regions ranging from higher‐order transmodal to lower‐order unimodal regions), which may have consequences for the relation to environment. This may imply the need to think about them in terms of their relation to the spatial interaction modes of the subject (i.e., disturbance of self/non‐self‐differentiation)
Ricci et al. (2025): *Modern perspectives on psychoses: dissociation, automatism, and temporality across exogenous and endogenous dimensions*	Narrative review (Article)	N/A	Exogenous (substance‐induced) and endogenous (schizophrenia) psychoses appear to differ phenomenologically in three ways: dissociation (distinct from spaltung), mental automatism (distinct from first‐rank symptoms), and temporality (via hyperpresentification rather than fragmentation). These differences are discussed in the light of current research and historical models.	**Sp:** Schizophrenia is characterized by a disturbance of internal time, which can disrupt the lived time and self, as well as autobiographical processes, and delusion is an attempt to reconstruct these various disturbances.
Delcourt, Englebert, and Pachoud (2025): *Bibliotherapy and Schizophrenia: a Stanghellinian Perspective*	Narrative review (Article)	N/A	Bibliotherapy is theoretically modelized as a person‐centered psychotherapeutic tool that may be suitable for cognitive, phenomenological, hermeneutic, and existential disturbances in persons with schizophrenia.	**Ds:** Minimal self‐disorders can be narratively and socially unfolded/described by the concerned person during clinical dialogue, or literarily, via bibliotherapy linked to certain expressions found in texts. This unfolding can give rise to a personal and social narrative and reflective re‐grasping of these disturbances of experience, which could fuel the process of personal recovery and retroactively influence these pre‐refective disturbances.
Jelsma et al. 2025: *The Association between Self‐Reported Self‐Disturbance Phenomena and Personal Recovery in Patients with a Schizophrenia Spectrum Disorder, Siblings and Controls*	Quantitative study (Article)	**Sample:** Patients with psychotic disorders (*n* = 522; 381 males; mean age = 33.83; 327 with schizophrenia, 97 with schizoaffective disorder, 48 with unspecified psychotic disorder, 19 with schizophreniform disorder, 10 with delusional disorder, 3 with substance‐induced psychotic disorders, 2 with psychotic disorders due to a medical condition), siblings (*n* = 608; 274 males; mean age = 34.24), control group (*n* = 369; 159 males; mean age = 38.44) **Aim:** To examine the association between self‐reported self‐disturbance phenomena and self‐reported personal recovery in patients, unaffected siblings and healthy controls **Measures:** PANSS, SELF, RAS‐24, GAF‐S, GAF‐F	Scores related to disturbances of the minimal self appear to be negatively correlated with those related to personal recovery in patients, siblings, and healthy controls. These correlations can be seen in the total scores and in scores related to some subdomains. Multiple hierarchical regression analyses show that minimal self‐disturbances (total and subdomain scores) partly explain the total and subdomain scores for personal recovery. These correlations appear to be independent of symptomatology	**Sp:** Minimal self‐disorders appear to impact the personal recovery of persons with schizophrenia spectrum disorder, either as a whole or in subdomains. This impact appears to be independent of symptomatology and may affect the reflective and autobiographical dimensions of the subject. The idea of a vulnerability spectrum (beyond diagnosis) is suggested

Abbreviations: ASD, Assessment of Self Descriptions; BPRS‐E, Brief Psychiatric Rating Scale‐Expanded; CASP, Critical Appraisal Skills Programme; Cbi, contextual model—biological aspect; CHIME, Connectedness, Hope, Identity, Meaning and Empowerment; Cso, Contextual model—social aspect; Cte, Contextual model—territorial aspect; DMN, Default Mode Network; Dp, Dialectical model—pathogenic trajectory; Ds, Dialectical model—salutogenic trajectory; EASE, Examination of Anomalous Self‐Experience; ENTREQ, Enhancing Transparency in Reporting the Synthesis of Qualitative Research; FEP, First Episode Psychosis; fMRI, Functional Magnetic Resonance Imaging; GAF, GAF‐S, GAF‐F, Global Assessment of Functioning, ‐Symptoms and ‐Function; MAIA‐2, Multidimensional Assessment of Interoceptive Awareness‐Version 2; MIN‐TAL, Montreal Neurological Institute templates' reported in Talairach atlas; NICE, National Institute for Health and Care Excellence; NSSE, Neurological Soft Signs Examination; PANSS, Positive and Negative Syndrome Scale for Schizophrenia; PRISMA, Preferred Reporting Items for Systematic reviews and Meta‐Analyses; RAS‐24, Recovery Assessment Scale—24 items; RETREAT, Review question, Epistemology, Time/Timescale, Resources, Expertise, Audience and purpose, Type of Data; SCID‐II PQ‐BPD, Borderline Personality Disorder items from the Structured Clinical Interview for DSM‐IV; SCI‐PANSS, Structured Clinical Interview for Positive and Negative Syndrome in Schizophrenia Scale; SELF, Self‐Experience Lifetime Frequency scale; SFS, Social Function Scale; SOC‐13, Sense of Coherence‐13 items; SOFAS, Social and Occupational Functioning Assessment Scale; Sp, Structural model—pathogenic trajectory; SPQ‐BR, Schizotypal Personality Questionnaire‐Brief Revised; Ss, Structural model—salutogenic trajectory; TAS, Tellegen Absorption Scale; TMT‐BA, Vocabulary subtest, Trail Making Test Parts A and B; TSCS‐2, Tennessee Self‐Concept Scale‐Second Edition, Short Form; UHR, Ultra‐High Risk; VISQ, Varieties of Inner Speech Questionnaire; WAIS‐IV, Wechsler Adult Intelligence Scale‐4th Version.

Regarding the quality assessment of theoretical and conceptual papers, including narrative reviews and case studies (which cannot be evaluated using CASP and are structured as narrative reviews), approximately two‐thirds of the QATTL items (i.e., assessment of writing, title, abstract, introduction, rationale/background, research questions, literature review, and findings), mainly relating to the clarity and argumentation of the papers, were generally of good quality^2^ (mean = 48.27%). However, slightly less than the remaining third of items (i.e., assessment of tables and figures, consideration of alternative frameworks, contextualization of discussion, strengths and limitations, emphasis on both positive and negative findings, and disclosures), primarily addressing concrete aspects and limitations of the argument, were either not applicable or of lower quality^3^ (mean = 44.35%).

Regarding the quality assessment of quantitative papers, all four cross‐sectional studies (*n* = 4) validated six of the 11 items on the CASP Cross‐Sectional Studies Checklist (2024a). Three studies validated four of the items, and two studies validated only one item (i.e., item 11, concerning the applicability of results to real‐world care contexts). For case–control studies (*n* = 2), both validated five of the 12 items on the CASP Case–Control Study Checklist (2024b). One study validated four items, and none validated three items (i.e., items 6b, 7, and 11, related to confounding factors, effect size, and alignment with other available evidence).

Regarding the quality assessment of qualitative papers, all studies validated the first two items of the CASP Qualitative Studies Checklist (2024c). Two studies validated approximately 60% of the items (i.e., appropriate research design, recruitment strategy, data collection, consideration of ethical issues, clear statement of findings, and overall contribution). Item 8 (i.e., rigor of data analysis) was validated by only one study, and none of the studies validated item 6 (i.e., adequate consideration of the relationship between researcher and participants).

Regarding their quality assessment, systematic reviews satisfied between 80% and 90% of the items on the CASP Systematic Review Checklist (2024d), with the shared exception of item 3, which pertains to the inclusion of all relevant papers for their respective research question.

The papers predominantly comprise theoretical perspectives, with few empirical studies. They are generally well‐structured and of moderately good quality but tend to be less robust regarding the applicability to real‐life contexts, the questioning or challenging of these links, or the generation of alternative explanatory or operational approaches. Based on this analysis, we identified three descriptive, non‐exclusive, cross‐cutting theoretical models and trends in the reviewed literature concerning how the minimal and narrative self in schizophrenia are linked. These trends have been synthesized as the three models detailed below. These models appear to be essentially tacit (i.e., not directly explored or formally presented as theoretical models), shared (i.e., emerging across diverse research communities), and oriented toward scientific thinking and practices (i.e., providing some conceptual models and operational structures). Notably, few studies directly or explicitly address the mechanisms linking these two levels of self. Additionally, terminology, definitions, and theoretical frameworks are largely inconsistent across studies. Therefore, the three models presented below are intended as preliminary, non‐exclusive conceptual frameworks to guide future theoretical and empirical research.

### 3.2 The Structural Model

**FIGURE 3:**
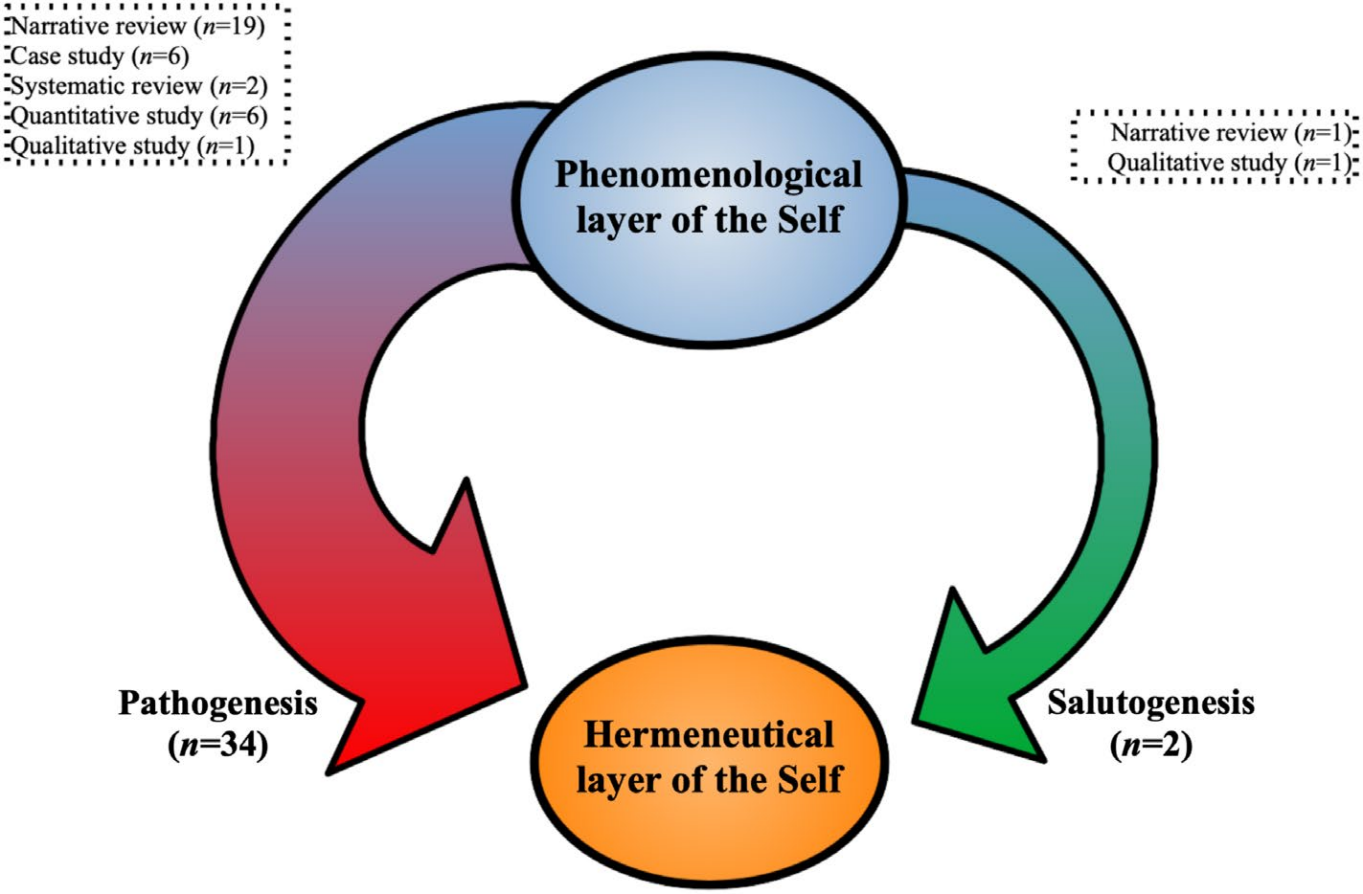
The Structural model.

The Structural model (Figure 3) appears to be the oldest and most consistently discussed model in the literature. This model proposes that narrative self‐disturbance (and the schizophrenia phenotype more broadly) is a secondary consequence of minimal self‐disturbance. As Parnas and Zandersen (2020) note, “disturbance of the structural level of selfhood, entailing an instability of the basic subject‐world relation, will also manifest as disturbance of narrative features, including interpersonal functioning, emotional regulation, and direction in life”, whereas “disturbance of the narrative level of selfhood will not in itself cause structural disorders of the core self”.

Moreover, we identified two trajectories within this Structural model: (1) what Antonovsky (1979, 1987; Mittelmark et al. 2022) termed *salutogenesis*, encompassing subjective, relational, and existential processes focused on health and adaptation, specifically the regaining of a sense of coherence when facing adversity; and (2) *pathogenesis*, emphasizing factors and processes underlying the development of a disorder.^4^ Within this structural perspective, the pathogenic trajectory, initiated by pre‐reflective self‐disturbances, is the most extensively discussed and is considered to generate symptoms—including delusions, hallucinations, autism, and psychosocial dysfunction—as well as disorders at the narrative level of the self, such as identity, life story, and metacognition (Ballerini 2004; Bilheran et al. 2009; Cermolacce et al. 2018; Chamond 1999; Charbonneau 2019; Cowan 2024; De Vries et al. 2013; Englebert 2021; Gallagher 2003; Green and García‐Mieres 2022; Heinz et al. 2012; Jelsma et al. 2025; Lysaker and Lysaker 2010; Mishara and Fusar‐Poli 2013; Mishara et al. 2013; Moe and Docherty 2014; Naudin et al. 2000, 2019; Nelson et al. 2013; Parnas and Henriksen 2019; Parnas and Zandersen 2020; Pienkos 2020; Poupart and Laigle 2024; Ricci et al. 2025; Ritunnano 2022; Ritunnano et al. 2022; Rosen et al. 2021; Sabbah and Northoff 2024a, 2024b; Simões 2023; Stanghellini et al. 2013; Svendsen et al. 2020; Yeh et al. 2024; Zandersen and Parnas 2019). Thus, symptoms and narrative disorders originate from disturbances of the pre‐reflective self and/or may mutually influence one another (e.g., delusions generated by basic self‐disturbance may alter personal identity; disrupted metacognitive processes may exacerbate delusions). Many interpretations suggest that primary disturbances in lived experience tend to fragment the subject's evolving identity and capacity for understanding, with symptoms emerging from this dual disruption. Additionally, recurring evidence indicates that disruptions in temporal experience and/or self‐affection may impair developmental processes through which events and identity are integrated into coherent continuity. Finally, the salutogenic trajectory, which is far less elaborated than the pathogenic trajectory, proposes that embodied and spontaneous interventions (e.g., art therapy, spatial‐corporeal mediations) may *act* at the pre‐reflective level to clinically influence pre‐reflective and reflective dimensions of the self toward personal and phenotypic implications (Englebert 2021; Mitchell and Meehan 2022).

In summary, this body of literature highlights the importance of understanding self‐disorders within the schizophrenia spectrum as arising from a structural‐hierarchical process, in which pre‐reflective disturbances of the self contribute both to (a) the manifestation of disorder‐related symptoms and (b) alterations in the narrative self.

### 3.3 The Dialectical Model

**FIGURE 4:**
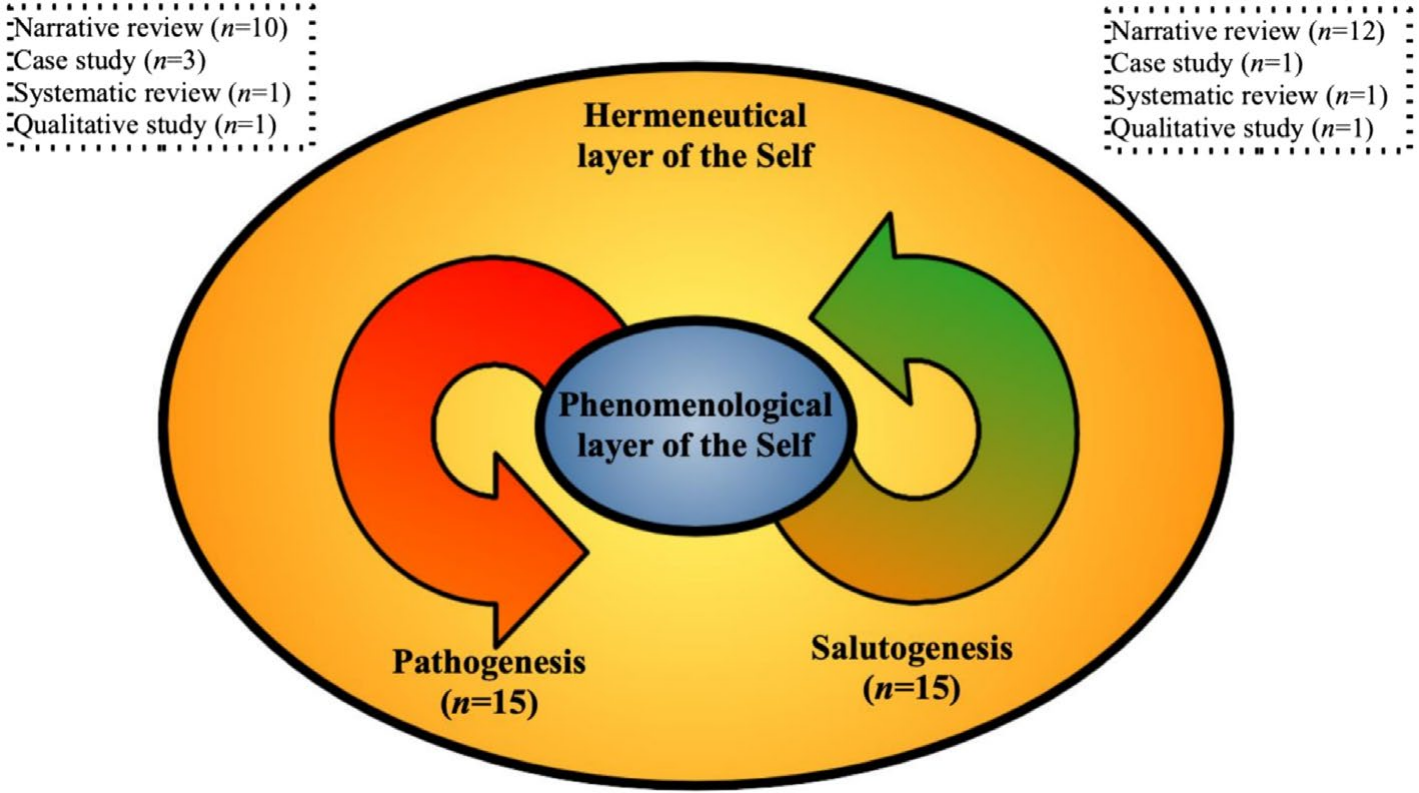
The Dialectical model.

The Dialectical model (Figure 4) appears to emerge as a response to the Structural model and its unilateral perspective, aiming to account for the bidirectional influences between the narrative and minimal self. Within this framework, pre‐reflective disturbances are considered consistently accessible to the individual through one's habitual mode of understanding oneself and one's lived experiences—that is, through one's tacit, active, and socially situated narrative positioning. As Stanghellini et al. (2023) note, “the person is at the same time a passive and active agent”, continually engaged in “a complex and dynamic interaction between anomalous changes in implicit aspects of experience” and two ongoing *self‐hermeneutical* activities: “(a) an implicit, pre‐reflexive and non‐deliberate ‘default’ level where basic experiences are shaped by the emotional and cognitive ‘common‐sense’ tools”, and “(b) an explicit, reflexive and deliberate position‐taking in front of these experiences, enacted as an active search for the meaning, cause, or reason for these basic experiences”.

The Dialectical model thus emphasizes the ongoing process of making meaning of personal experiences, which does not occur solely within the basic or narrative self, but rather unfolds in dialogue between different aspects of the self and between the self and others. This model incorporates both salutogenic and pathogenic trajectories. The pathogenic trajectory appears to function similarly to the structural pathogenic trajectory at the phenotypic level. In this context, symptoms and related psychosocial dysfunctions are thought to arise from a distress‐generating disturbance in reflective‐narrative positioning, affecting how one understands and *lives‐with* pre‐reflective disorders. Simultaneously, this disturbance also influences the pre‐reflective level of the self, producing basic experiences of perplexity and loss of common sense (Berkhout et al. 2019; Bilheran et al. 2009; Chamond 1999, 2017; Cermolacce et al. 2018; Koenig 2017; Lobaccaro 2022; Mishara and Fusar‐Poli 2013; Mishara et al. 2013; Naudin et al. 2000; Pienkos 2020; Poupart and Laigle 2024; Ritunnano et al. 2022; Stanghellini et al. 2013; Stanghellini et al. 2023). Within this model, meaning is frequently placed *before* perception in the genesis of lived experience, and the literature recurrently highlights either the possibility of a meta‐cognitive disorder or a more embodied, lived process of meaning‐making. Consequently, the pre‐reflective level of the self appears continuously engaged in a dialectical interaction with hermeneutic self‐reflection, which may, in turn, generate pre‐reflective disturbances. Finally, the salutogenic trajectory is oriented toward personal recovery—without discounting symptomatic or functional improvements—and appears to involve dimensions of identity, existential orientation, and social reintegration (Cermolacce et al. 2018; Delcourt, Englebert, and Pachoud 2025; Green and García‐Mieres 2022; Jimenez and Green 2024; Koenig 2017; Mishara et al. 2013; Naudin et al. 2019; Pienkos 2020, 2024; Poupart and Laigle 2024; Ritunnano 2022; Ritunnano et al. 2022; Stanghellini et al. 2013; Stanghellini et al. 2023; Troubé 2021).

In summary, this body of literature underscores the hermeneutic, existential, and *personal* dimensions of individuals' lived experience, moving beyond purely *transcendental* considerations, both in the development of disorders and in the recovery process.

### 3.4 The Contextual Model

**FIGURE 5:**
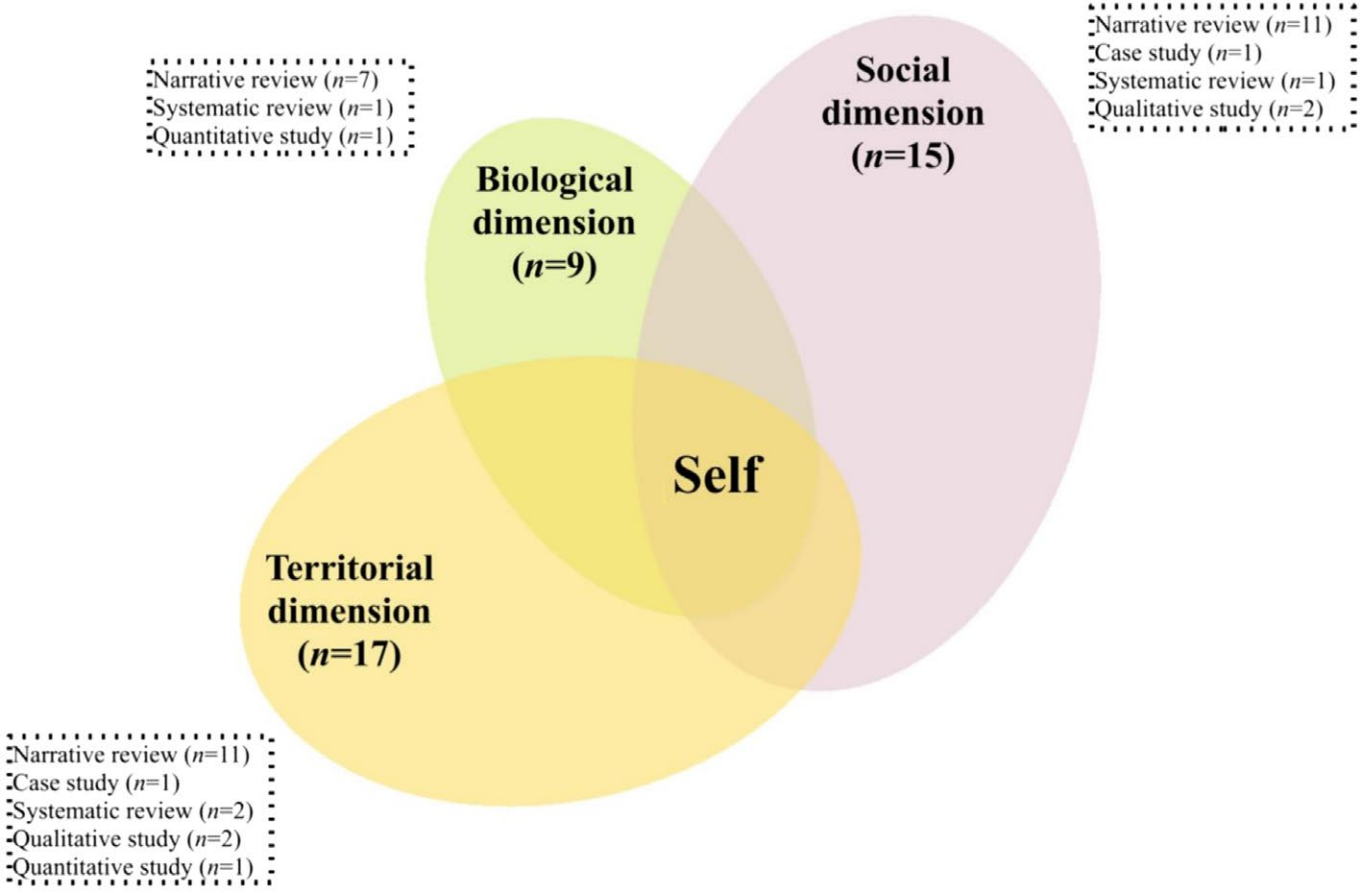
The Contextual model.

Compared to earlier models, the Contextual model (Figure 5) is more recent and distinguished by its emphasis on and elaboration of the self‐world relationship. As Pienkos (2020) observes, “while we can look to the subject to see how he or she constitutes or experiences the world, this does not imply that the origins of these experiences are to be found solely *within* the subject; instead they arise from a complex system of subject‐world”, highlighting the need “for a more contextual approach to the phenomenology of schizophrenia”.

From this perspective, the included studies emphasize three contextual domains in which the self is always already engaged: socially (interpersonally, clinically, culturally, politically), territorially (environmentally, bodily, in daily life), and biologically (neurologically, neurocognitively).

The social domain refers to the notion that the clinical phenotype, as well as pre‐reflective and narrative disturbances in schizophrenia, may arise from relational rupture or disharmony, while recovery may involve reciprocal rehabilitation of the connections between the individual, other persons, and one's socio‐cultural world (Ballerini 2004; Berkhout et al. 2019; Cermolacce et al. 2018; Green and García‐Mieres 2022; Heinz et al. 2012; Koenig 2017; Lobaccaro 2022; Naudin et al. 2019; Pienkos 2020, 2024; Ritunnano 2022; Ritunnano et al. 2022; Simões 2023; Stanghellini et al. 2013, 2023).

The territorial domain, sometimes also referred to as *ecological*, denotes that these disturbances may emerge from a disruption of rhythm, sociality, flexibility, and the taken‐for‐granted aspects of *everyday* life as a tacit condition of lived experience (Berkhout et al. 2019; Englebert 2021; Green and García‐Mieres 2022; Koenig 2017; Lobaccaro 2022; Naudin et al. 2019; Pienkos 2020, 2024; Ritunnano et al. 2022; Stanghellini et al. 2013, 2023; Troubé 2021). Likewise, these disturbances may arise from the interrelation between individuals and their immediate environment, encompassing the relationships among one's body, surrounding space, other persons' bodies, and the presence of objects that mediate lived experience (Berkhout et al. 2019; Cermolacce et al. 2018; Englebert 2021; Lobaccaro 2022; Mishara and Fusar‐Poli 2013; Naudin et al. 2019; Pienkos 2020; Ritunnano et al. 2022; Sabbah and Northoff 2024a, 2024b; Troubé 2021; Yeh et al. 2024).

The biological domain^5^ refers to the hypothesis that self‐disturbances are associated with specific neural correlates, including higher‐order transmodal and lower‐order unimodal regions, dopaminergic neurotransmission, default mode, limbic, and cortico‐subcortical networks, as well as the temporoparietal junction. These neural correlates are proposed to contribute to the emergence of aberrant salience and attributions, a disrupted self/non‐self distinction, and a vivid imagination that may attenuate the individual's engagement withp environment (Benítez‐Burraco et al. 2023; Cowan 2024; Jimenez and Green 2024; Lobaccaro 2022; Mishara and Fusar‐Poli 2013; Mishara et al. 2013; Rosen et al. 2021; Sabbah and Northoff 2024a, 2024b).

In summary, this body of literature underscores the importance of conceptualizing self‐disorders in schizophrenia from a contextual perspective, in which all levels of the self and dimensions of self‐disturbances continuously interact with the individual's interpersonal and socio‐cultural world, physical and everyday environment, and neural and cognitive correlates.

## 4 Discussion

Our central research question—*What are the links between the minimal and narrative dimensions of the self in the schizophrenia spectrum?*—was seldom addressed directly or explicitly in the included literature. Most studies developed theoretical arguments, occasionally supplemented by data from a small number of clinical cases, concerning the relationship between these two levels of selfhood. In many instances, this relationship was not the primary research aim; it was mentioned in passing while the focus remained on other psychopathological constructs, therapeutic processes, or differential diagnosis. Overall, these links appeared to be largely theoretical, tacit, collectively shared, and oriented toward some existing scientific models and practices.

In fact, we did not identify any studies that empirically examined the various recurring identified mechanisms underlying these links using comprehensive methods and both meaningful and adequate sample sizes. Moreover, based on the quality assessment of the included papers, future research should not only investigate the relationship between the pre‐reflective and reflective self more thoroughly in empirical, real‐world contexts—even within theoretical studies—but also critically examine the assumptions and limitations of this link. This is particularly important because alterations of narrative identity have been proposed as a transdiagnostic process in mental illness (Cowan and Lind 2024; Thomsen, Cowan, and McAdams 2025); consequently, certain aspects of narrative self‐disturbance may not be specifically coupled to the psychopathology of schizophrenia spectrum disorders. In this integrative review, we have described three models that illustrate key tendencies in the tentative literature on this potential link. It is important to note that these models are not mutually exclusive, and their theoretical diversity appears to reflect the richness of the research question rather than insurmountable doctrinal divides. Indeed, the pre‐reflective level, central to the Structural model, cannot be understood independently of the world and the contextual embedding of the self (Zahavi 2020). Clinical disturbances of selfhood involve a disorder of the minimal self as it manifests in perception, thought, imagination, and ante‐predicative experience of others and the world (Parnas and Sandsten 2024; Rasmussen 2024; Sass and Parnas 2003). In other words, these models do not represent successive *revolutions* or paradigm shifts, even if some studies claim otherwise—particularly since such a *revolutionary* view (i.e., to abandon one approach entirely in favor of another) may compromise the necessary integrative complexity in psychiatry and psychopathology (Kendler 2005; Stein et al. 2022, 2024). Notably, the social and territorial phenomena highlighted in the Contextual model may further articulate and substantiate disturbances of the self‐world relation, which are also central to the Structural (Parnas et al. 2002) and Dialogical models (Lysaker, Kukla, et al. 2020; Lysaker, Minor, et al. 2020). Moreover, across the Structural, Dialogical, and Contextual models, there is a clear progression from emphasizing the *passivity* of self‐processes toward acknowledging their *activity*.

### 4.1 Toward Self's *Activity*

The three reviewed models indicate an increasing focus on the *activity* of self‐processes and explore the extent to which the individual can *act* within their own situation and developmental trajectory. This activity encompasses both conscious, reflective efforts and habituated or pre‐reflective processes. Studies addressing *salutogenesis* particularly highlight activity within subjectivity and personal recovery, aligning with contemporary clinical approaches that emphasize individuals' lived experience and agency (Lysaker and Hasson‐Ohayon 2021; Lysaker and Klion 2017; Roe et al. 2021; Stanghellini 2019). Emphasizing activity in the self‐constitution of the individual opens not only new theoretical perspectives but also, crucially, clinical ones. For instance, while the psychopathology literature has traditionally described pre‐reflective experiences (e.g., thought pressure), future research should also examine how individuals act upon these experiences—pre‐reflectively and/or reflectively—through habits, objects, and behaviors (Englebert and Cermolacce 2024). This approach is consistent with ongoing developments in the dialogue between psychiatry and philosophy (Stein et al. 2024). The notion of self‐activity, both reflective and pre‐reflective, aligns with Sartre's (1936) claim that “transcendental consciousness is an impersonal spontaneity”. As Gallagher (2000, 2004) has discussed, the minimal self embodies an intrinsic sense of agency, evident in the individual's continual engagement with oneself and the world, while the narrative self enacts motivations to achieve agentic and communal goals and to intentionally organize self‐understanding (Conway and Pleydell‐Pearce 2000; Habermas and Bluck 2000; McAdams 1988; McLean et al. 2007; Zahavi 2008).

Activity is particularly emphasized in the Dialectical model. One perspective within this model conceptualizes self‐disturbances as a disrupted dialogue between the individual and their lived experience, in which the narrative self negotiates and integrates multiple self‐positions in relation to others and the world (Lysaker et al. 2012). It is proposed that individuals who struggle to derive meaning from their lived experience may experience increasing fragmentation in the experiential field, with events losing their connections to others (Cowan et al. 2021; Lysaker et al. 2022). The Contextual model, by contrast, highlights engagement with the world through socio‐ethological negotiating processes. Personal recovery also aligns with this emphasis on activity in establishing or reconstructing identity. Consequently, future research—consistent with recent proposals by Sass and Feyaerts (Feyaerts and Sass 2023; Sass and Feyaerts 2023)—should investigate the pre‐reflective and clinical dimensions of this activity, always in dialogue with lived experience, meaning, and the surrounding environment.

Moreover, this emphasis on activity resonates with the history of psychiatric phenomenology, initiated in 1922 by Binswanger and Minkowski (Delcourt, Auxenfants, et al. 2025), particularly in the latter's work. A central question remains: to what extent and by what mechanisms can pre‐reflective disorders of selfhood be mediated or compensated by contextual or reflective processes? This issue remains largely unexplored, but Minkowski (1927, 1933) introduced a concept that appears especially fruitful: *phenomenological compensations*. These refer to various tacit processes—reflective, imaginative, emotional, and behavioral—that enable an individual to *live‐with* a disorder arising from a primary illness process, similar to the mechanisms described in the models discussed here (Urfer 2001; Urfer‐Parnas 2018). Such *compensations*, including morbid rationalism or exaggerated fantasy, attempt to “fill the void” created by schizophrenic autism (i.e., the pre‐reflective loss of vital contact with reality). They are characterized as repetitive structures observed with detachment from pragmatic concerns, reflecting the underlying deficit in attunement with others and the world, while simultaneously striving to maintain engagement with it. In Minkowski's view, these processes do not fundamentally cure the primary illness, but they may mitigate its consequences. From our perspective, this active approach warrants renewed theoretical and clinical consideration and aligns with current developments in contemporary literature.

### 4.2 Beyond Self's *Narrativity*

This literature also reveals a theoretical trend that warrants attention. The link between the minimal and narrative self in the schizophrenia spectrum appears largely to be a matter of philosophical or theoretical propositions that remain to be empirically and clinically explored. This gap between theory and empirical work highlights the challenges of applying philosophical frameworks to real‐world contexts (Abettan 2015). Future research would benefit from more comprehensive clinical‐phenomenological investigations, as exemplified by original studies on pre‐reflective self‐disorders (Nordgaard et al. 2021), as well as qualitative, first‐person, interdisciplinary, and mixed‐methods approaches that distinguish these two levels of selfhood and provide insights into their interrelations over time and across clinical interventions (Kyzar and Denfield 2022; Zieber and Wojtowicz 2019). Additionally, recent neuroscientific developments suggest a growing need for methodological diversity, emphasizing the importance of empirical investigations into these links.

One important motivation for this approach is to investigate these levels of self as both *given* and *active* at a pre‐verbal level, within pre‐reflective and pre‐narrative perspectives. It is proposed that subjective disturbances—assessed using semi‐structured instruments such as the Examination of Anomalous Self‐Experience (Parnas et al. 2005) and related scales (Bizzari et al. 2025; Rasmussen et al. 2018; Sass et al. 2017)—can also be examined through a pre‐narrative empirical and neuroscientific lens, across visual, auditory, tactile, or multisensory modalities (Di Cosmo et al. 2021; Foucher et al. 2007; Giersch et al. 2008; Martin et al. 2013; Noel et al. 2018; Schmidt et al. 2011; Stevenson et al. 2017). In particular, this can be achieved through tasks assessing temporal asynchrony between the onset of two stimuli. Temporal processes constitute a fundamental aspect of perceptual experience, recognized in both empirical (Herzog et al. 2020; VanRullen 2016, 2018) and philosophical traditions (Husserl 1928; Ricoeur 1985). Disturbances in these temporal processes are observable at the perceptual level, independent of language, for example as difficulty discriminating events in time (Arrouet et al. 2022; Capa et al. 2014; de Boer‐Schellekens et al. 2014; Foerster et al. 2021; Fuchs 2007; Lalanne et al. 2010, 2012; Marques‐Carneiro et al. 2021; Minkowski 1933; Stanghellini et al. 2015; Vogeley and Kupke 2006). Timing disorders have also been reported in autobiographical memory, future projection, working memory, and long‐term memory (Allé et al. 2016; Ben Malek et al. 2019; Dreher et al. 2001; Landgraf et al. 2011; Liu et al. 2020; Schwartz et al. 1991). Employing these experimental tools could therefore help bridge the gap between the minimal and narrative self in future research (e.g., see Piani et al. 2025), particularly given that this domain pertains to the life of the self and that self‐disorders may impact multiple linguistic and expressive processes (Cowan and Allé 2026; Kolaiti 2019).

In the Contextual model, territorial explorations, grounded in social and everyday dimensions, warrant greater attention in future research, as they remain relatively underdeveloped. The social dimension of the Contextual model appears to require not only further specification but also reconsideration analogous to that applied to activity. While some authors have called for a *socialization* of the minimal self (Bower 2023; Kyselo 2016; Gallagher 2023), this perspective appears more directly relevant to the narrative self, given that sociocultural relations play a central role in the development and sustenance of narrative identity (Haden et al. 1997; McAdams 2001; Thomsen, Fivush, et al. 2025). Individuals living with psychosis have highlighted the need to emphasize meaning‐making processes that resonate with their lived experiences, even when these diverge from dominant sociocultural narratives (Cowan and Allé 2026; Ingall et al. 2025; McLean and Syed 2016; Thomsen et al. 2023). For example, some individuals may be receptive to mystical or supernatural interpretations of unusual experiences, which may provide comfort, despite not aligning with psychiatric definitions of psychosis or with any models discussed in this review. Future research should examine efforts to frame lived experience through unconventional or non‐normative meanings that nonetheless may support personal recovery.

### 4.3 Clinical Perspectives

Firstly, the Structural model appears to align with a psychiatric perspective. Its focus on pathogenesis rather than salutogenesis reflects a focus on symptoms or related factors (e.g., insight, cognition) commonly observed in psychiatric practice, from which therapeutic considerations can subsequently be developed. Because it argues for a structure in terms of primary and secondary levels in the pathogenesis of schizophrenia, it is potentially amenable to empirical testing in longitudinal studies (comparable to the studies of self‐disorders as predictors of subsequent development of psychosis (Nelson et al. 2012; Raballo et al. 2025)). However, this model provides limited operational guidance for professionals, family members, peer support, and so forth, in concrete clinical interpersonal contexts (e.g., clinical programs or stances/postures).

Secondly, the Dialectical model aligns more closely with a psychotherapeutic perspective. Its relative balance between salutogenic and pathogenic trends reflects both its theoretical intent and its practical capacity to combine understanding of the genesis of the disorder's subjective manifestations with opportunities for individuals to act upon these experiences, situating the disorder within psychotherapeutic and recovery‐oriented frameworks (Haram et al. 2019; Rossi et al. 2022; Ruffalo 2023). The main strength of this model is its ability to actively involve the individual in understanding and coping with the subjective manifestations of the disorder. Nevertheless, this model also presents limitations, such as a lack of operationalization that hinders clarification of causal chains and associated factors in the therapeutic relationship and personal coping strategies, which may limit integration with structured interdisciplinary approaches.

Lastly, the Contextual model represents a more heterogeneous perspective. Unlike the other models, it is still too new and underdeveloped to clearly show salutogenic and/or pathogenic trends. Its strength lies in its openness to growing interdisciplinarity in contemporary approaches (Barlati et al. 2022; Fuchs 2018; Gauld and Micoulaud‐Franchi 2022; Slimmen et al. 2024)—particularly ecological and psychosocial perspectives concerned with individuals' daily and material living conditions—which may have subsequent clinical relevance. This model shifts focus from an intra‐subjective perspective toward diverse extra‐subjective environmental approaches, while maintaining dialogue with neuroscientific data and paradigms. Its limitations arise from a lack of internal theoretical and operational consistency, reflecting the challenge of linking minimal and narrative self to the pluralistic elements shaping the individual's environment and interactional dynamics.

Taken together, these considerations suggest that understanding the links between minimal and narrative self in the schizophrenia spectrum involves not only conceptualizing how the disorder develops or is coped with, but also recognizing specific clinical orientations and interventions. Future research should therefore enrich and integrate pluralistic clinical approaches, accommodating both consistency and divergence from existing models.

### 4.4 Limitations

A clear limitation of this research literature is the lack of standardization in terms and concepts. Certain terms (e.g., minimal self) have conventionally recognized, bibliographically rooted definitions (e.g., Bovet and Parnas 1993; Cermolacce et al. 2007; Gallagher 2000; Sass and Parnas 2003), although the correspondence between their philosophical and clinical definitions has been questioned (Carruthers and Musholt 2018). Other terms (e.g., narrative self) lack similarly established bibliographic definitions and have unclear relationships to associated concepts (e.g., autobiographical process, social identity, self‐reflection). This variability has affected our cross‐analysis and is likely to pose challenges for future operational research. Indeed, such diversity appears to have contributed to an expansion of theoretical proposals at the expense of empirical investigation and modeling.

Another limitation concerns the limited number of studies exploring the mechanistic relationship between the minimal and narrative self in schizophrenia. This limitation is compounded by the exclusion of studies that attempt to integrate these two levels of selfhood, particularly those employing qualitative methods aimed at capturing lived experience through personal meaning. As noted by Ritunnano et al. (2022), there is “a lack of dedicated qualitative research methodologically tailored to capture alterations of minimal selfhood [due to] the inherent ineffability of self‐disturbances, and their limited accessibility to language”. This constraint has also led to the exclusion of several studies addressing the *sense of self*, which, although conceptually enriching, often do not distinguish between levels of selfhood and implicitly assume the links under investigation. Consequently, the various mechanisms identified repeatedly in the literature are worthy of further empirical and theoretical explorations. Future research would benefit from maintaining a clear distinction between levels of self and types of disorder, while employing appropriate operationalized, mixed‐methods protocols and models.

## 5 Conclusion

Few studies have been primarily dedicated to investigating the potential links between the minimal and narrative self in the schizophrenia spectrum. Contrary to our expectations, these links have not been the focus of direct, causal, or operational studies and modeling; most papers discuss them only in relation to other research questions. Furthermore, this literature appears largely theoretical and occasionally speculative rather than empirical. Some of the constructs involved are inconsistently defined, highlighting the need for greater conceptual clarity and mechanistic exploration (i.e., how does those links concretely works?) in future research.

From this perspective, three non‐exclusive models appear to frame current approaches to understanding these links: (1) disorders of the minimal self structurally influence narrative self and the schizophrenia phenotype, with a pathogenic focus; (2) disorders of the narrative self exert dialectical retroactive effects on minimal self‐disturbances, contributing to the schizophrenia phenotype, with a salutogenic focus; and (3) self and its disorders are primarily contextually related.

Although this corpus remains preliminary, it opens broad avenues for discussion and research on the self in psychiatric care, engaging diverse research communities and perspectives (e.g., Feyaerts and Sass 2025; Raballo et al. 2025; Sass et al. 2018). As Spencer et al. (2024) noted, “the future of phenomenological psychopathology requires further heterogeneity”, and the trends identified in this integrative review offer highly generative pluralistic and integrative perspectives that may stimulate both theoretical development and, particularly, future empirical research.

## Author Contributions

**Florestan Delcourt:** conceptualization (equal), data curation (equal), formal analysis (equal), methodology (lead), software (lead), supervision (lead), validation (equal), writing – original draft (lead), writing – review and editing (lead). **Henry R. Cowan:** conceptualization (equal), data curation (equal), formal analysis (equal), methodology (equal), validation (equal), writing – review and editing (equal). **Jordan Sibéoni:** conceptualization (equal), data curation (equal), formal analysis (equal), methodology (equal), validation (equal), writing – review and editing (equal). **Mélissa C. Allé:** conceptualization (equal), data curation (equal), formal analysis (equal), methodology (equal), validation (equal), writing – review and editing (equal). **Andreas C. R. Rasmussen:** conceptualization (equal), data curation (equal), formal analysis (equal), methodology (equal), validation (equal), writing – review and editing (equal). **Rosa Ritunnano:** conceptualization (equal), data curation (equal), formal analysis (equal), methodology (equal), validation (equal), writing – review and editing (equal). **Anne Giersch:** conceptualization (equal), validation (equal), writing – review and editing (equal). **Fabian Lo Monte:** conceptualization (equal), data curation (equal), formal analysis (equal), methodology (equal), validation (equal), writing – review and editing (equal). **Jérôme Englebert:** conceptualization (equal), data curation (equal), formal analysis (equal), methodology (equal), validation (equal), writing – review and editing (equal). **Bernard Pachoud:** conceptualization (equal), data curation (equal), formal analysis (equal), methodology (equal), validation (equal), writing – review and editing (equal).

## Funding

The authors have nothing to report.

## Conflicts of Interest

The authors declare no conflicts of interest.

## Related WIREs Articles

Consciousness Under the Spotlight: The Problem of Measuring Subjective Experience

The Multiple Dimensions of Familiarity: From Representations to Phenomenology

## Data Availability

Data sharing is not applicable to this article as no new data were created or analyzed in this study.

## Endnotes

^1^
Je pense que je pense et d'y penser je suis
*Et ne suis parce que je pense*

*Et ce triste savoir embrouille ma science*

*Ajoute une nuit à mes nuits.*

*Que ne suis‐je sans être et sans une mémoire*

*Mêlant le demain et l'hier*

*Et qui déroule en moi cette mouvante moire*

*Dangereuse comme la mer.*

*Mieux me va le sommeil et son vague mélange*

*Où je ne me charge de rien*

*Et son théâtre obscur dont la pièce me change*

*En un moi qui n'est plus le mien.*

^2^

*partially* item mean = 30.35%; *completely* item mean = 15.77%.

^3^

*mostly* item mean = 22.91%; *partially* item mean = 22.46%; *completely* item mean = 5%.

^4^
In this work, the distinction between *salutogenesis* and *pathogenesis* does not follow the clinical and ethical considerations of Antonovsky, but the distinction he makes between therapeutic theoretical thinking centered on personal evolution and centered on the disorder's course.

^5^
We chose to situate the biological domain—so named due to the diversity of its neuroscientific foundations and in contrast to the intra‐subjectivist orientation of the two preceding models—within the Contextual model, since, as Fuchs (2018) emphasizes, the brain is distinct from the person and is not an organ centered on the subject shaping its interiority, but rather “an organ of mediation, transformation, and modulation, being embedded in the human organism's relationships with the surrounding world and in interpersonal relationships”, reflecting a context‐focused perspective.
